# Anthracene‐Fused Oligo‐BODIPYs: A New Class of π‐Extended NIR‐Absorbing Materials

**DOI:** 10.1002/anie.202214543

**Published:** 2022-12-08

**Authors:** Jorge Labella, Gonzalo Durán‐Sampedro, Swathi Krishna, M. Victoria Martínez‐Díaz, Dirk M. Guldi, Tomás Torres

**Affiliations:** ^1^ Department of Organic Chemistry Universidad Autónoma de Madrid Campus de Cantoblanco C/Francisco Tomás y Valiente 7 28049 Madrid Spain; ^2^ Department of Chemistry and Pharmacy Interdisciplinary Center for Molecular Materials (ICMM) Friedrich-Alexander-Universität Erlangen-Nürnberg Egerlandstr. 3 91058 Erlangen Germany; ^3^ Institute for Advanced Research in Chemical Sciences (IAdChem) Universidad Autónoma de Madrid 28049 Madrid Spain; ^4^ IMDEA—Nanociencia C/Faraday 9, Campus de Cantoblanco 28049 Madrid Spain

**Keywords:** BODIPY, Conjugation, Dyes/Pigments, NIR, π-Systems

## Abstract

Large π‐conjugated systems are key in the area of molecular materials. Herein, we prepare via Au^I^‐catalyzed cyclization a series of fully π‐conjugated anthracene‐fused oligo‐BODIPYs. Their structural and optoelectronic properties were studied by several techniques, ranging from X‐ray, UV/Vis, and cyclic voltammetry to transient absorption spectroscopy. As a complement, their electronic structures were explored by means of Density Functional Theory (DFT) calculations. Depending on the size and shape of the π‐conjugated skeleton, unique features—such as face‐to‐face supramolecular organization, NIR absorption and fluorescence as well as strong electron accepting character—were noted. All in all, the aforementioned features render them valuable for technological applications.

## Graphical abstract

Au^I^‐catalyzed cyclization has been used to prepare a series of fully π‐conjugated anthracene‐fused oligo‐BODIPYs. The size and shape of the π‐conjugated skeleton determines the unique features of the compounds—such as their face‐to‐face supramolecular organization, NIR absorption and fluorescence as well as strong electron accepting character.

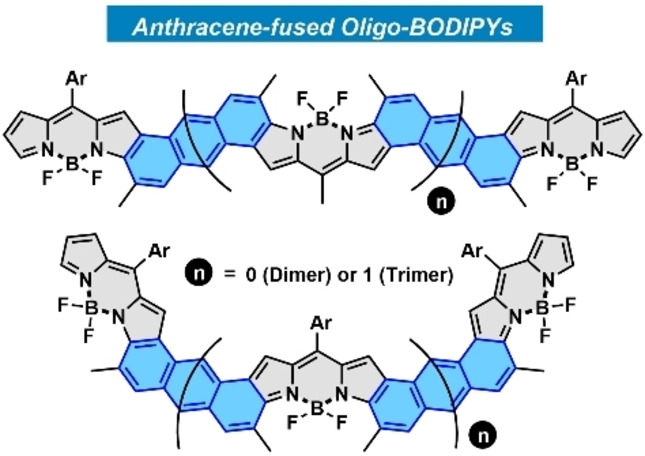

## Introduction

Exploration of polycondensed π‐systems is currently one of the hot topics in molecular materials, since they are promising candidates for multiple applications including semiconductors, molecular wires, or near‐infrared absorbers.[^1^, ^2^] Among these, oligomeric (e.g., dimers or trimers) porphyrinoid‐based systems have been extensively studied for the last decade. These derivatives possess unique optoelectronic properties, which arise from an effective π‐delocalization.^[3]^ In this context, porphyrins (Pors) have been at the forefront of investigations as their peripheral and meso positions are highly reactive and, as such, the aromatic structure is readily enlarged by means of numerous reactions.^[4]^ A variety of fully conjugated oligo‐porphyrins of different size and shape have been synthesized.[^5^, ^6^] To a lesser extent, polybenzenoids based on phthalocyanines (Pcs),^[7]^ corroles (Cor)^[8]^ or even subphthalocyanines (SubPcs)^[9]^ have also been reported. A wide range of potential applications have been demonstrated. But, at the same time, they are increasingly unstable when increasing the π‐system. Responsible is a HOMO, whose energy levels is significantly raised. In addition, large porphyrinoids often are subject to aggregation, which renders their synthesis and purification a tedious task. This opened the search for alternative chromophores as building blocks for highly π‐conjugated systems. Higher stabilities and better solubility are essential criteria for the versatility and further development of this field.

**Figure 1:**
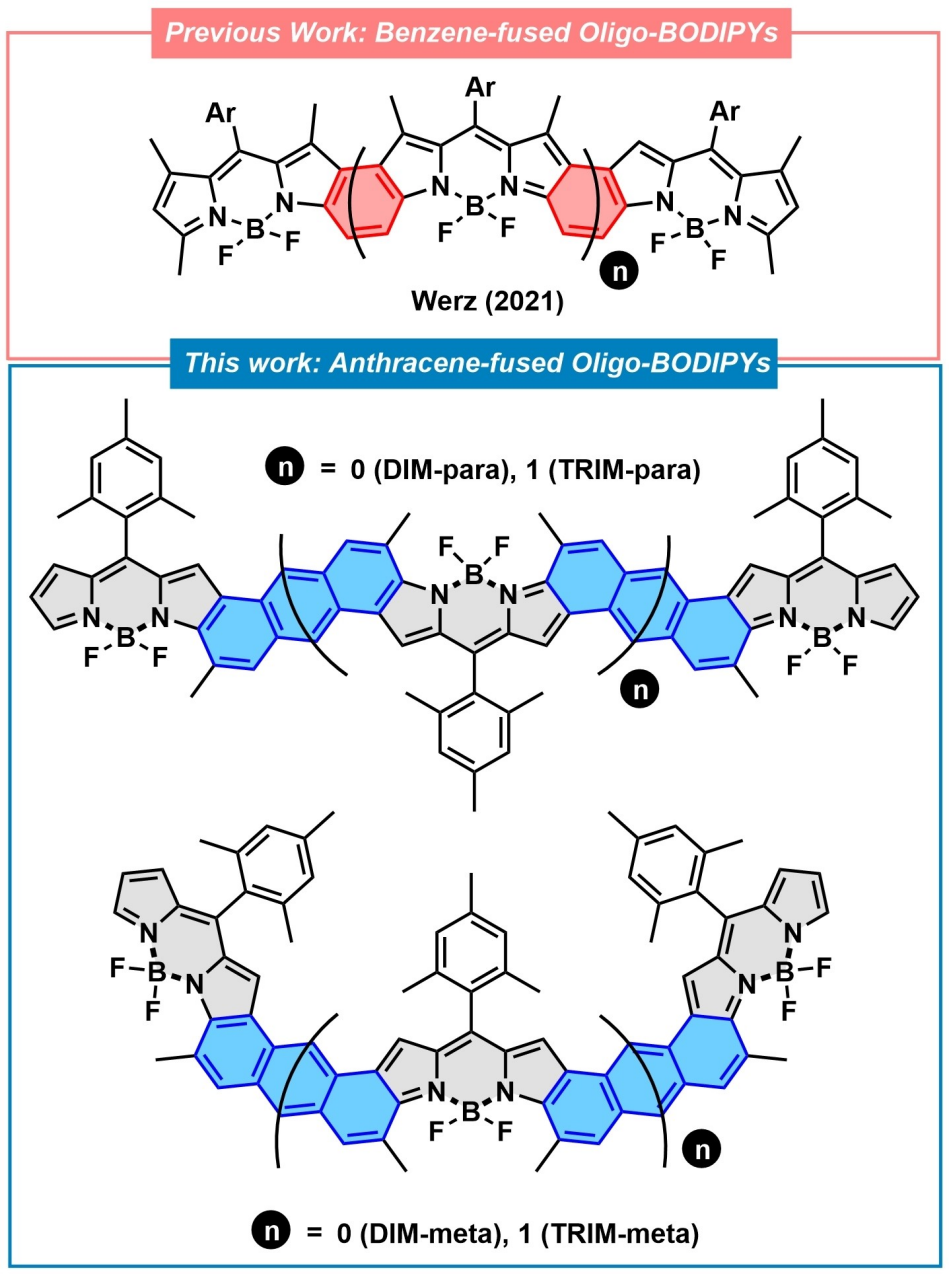
Previously reported benzene‐fused oligo‐BODIPYs (top) and the anthracene‐fused Oligo‐BODIPYs reported in this work (bottom).

BODIPYs (boron dipyrromethenes), which are structurally related to porphyrins, are a well‐known class of chromophores. They are based on a fully π‐conjugated pyrrole‐based scaffold and a tetrahedral boron atom, which provide excellent solubility and stability.^[10]^ Similar to cyclic porphyrinoids, the pyrrole rings of BODIPY are post‐functionalizable with different substituents. Important is hereby the conjugation, which strongly modulate the optoelectronic properties of the whole system. As a matter of fact, BODIPYs have made it into multiple research areas, such as organic photovoltaics,^[11]^ OLEDs,^[12]^ or photodynamic therapy.^[13]^ Still, the use of BODIPYs to prepare large π‐fused oligomers remains a rather unexplored field. Such a scarcity is ascribed to the limited synthetic methods for the preparation of fused BODIPYs beyond monomeric species. In this regard, only a handful of examples of non‐monomeric π‐extended BODIPYs have been reported. Notable, BODIPYs are linked by a single benzene at their alpha or beta positions.^[14]^ For instance, Werz and co‐workers have recently reported an innovative method to prepare a series of benzene fused oligo‐BODIPYs with strong NIR responses and interesting redox properties (Figure 1; top).^[15]^ Other examples include the use of aromatic spacers, which are, however, non‐effective in terms of conjugation. The formation of Clar′s π‐sextets is the bottleneck.^[16]^ In light of these precedents, it is of great interest to develop π‐extended oligo‐BODIPYs with larger hydrocarbons such as naphthalene or anthracene as spacers that ensure an effective conjugation across the whole π‐scaffold.

**Scheme 1:**
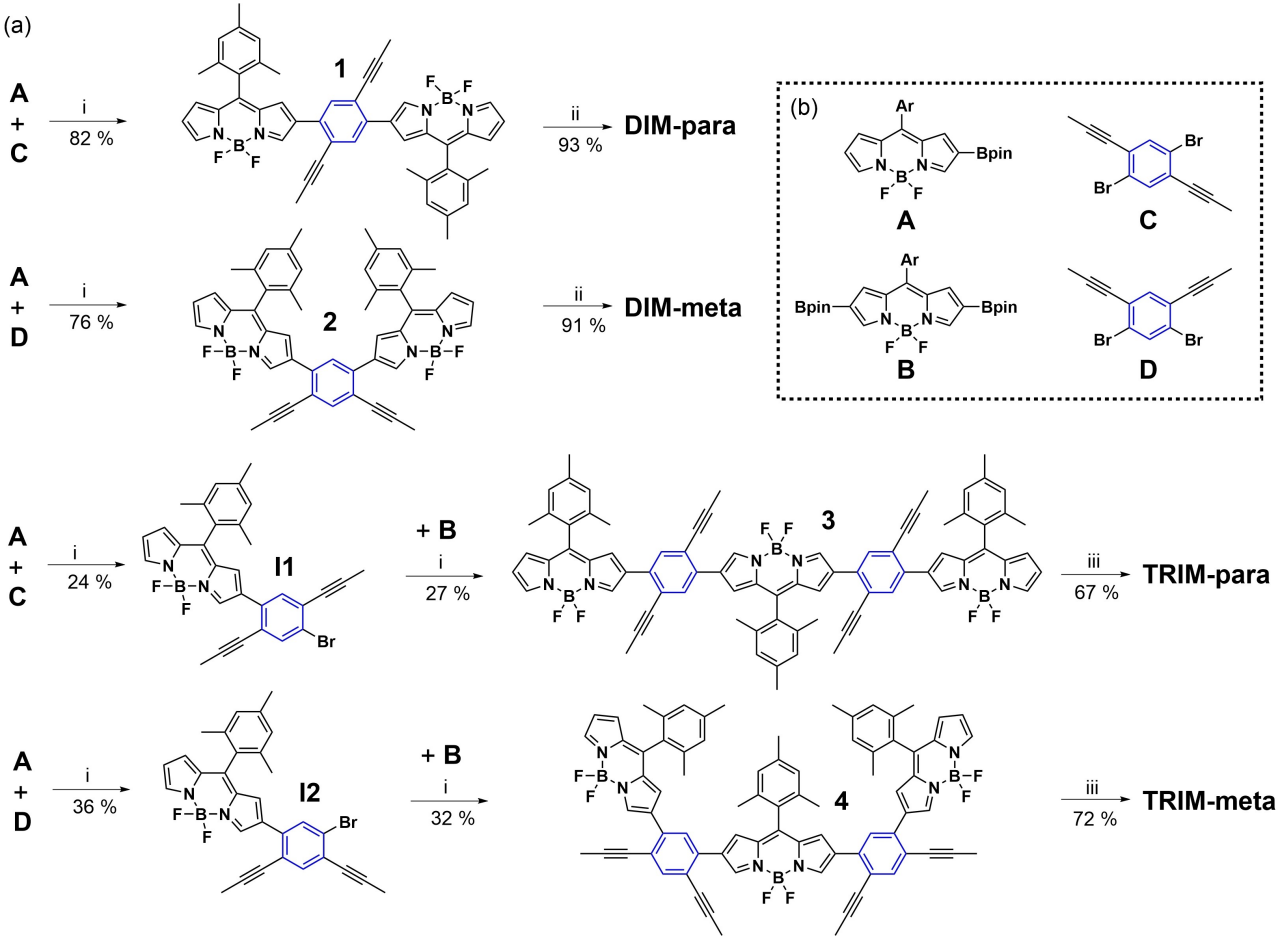
a) Synthetic strategy towards BODIPY‐anthracene oligomers; ^*a*^ b) Structure of the key building blocks. ^*a*^ Reagents and conditions: (i) [(2‐Di‐*tert*‐butylphosphino‐2′,4′,6′‐triisopropyl‐1,1′‐biphenyl)‐2‐(2′‐amino‐1,1′‐biphenyl)]palladium(II) methanesulfonate, K_3_PO_4_, THF/H_2_O, r.t, 10 min; (ii) PPh^F^_3_AuCl, AgSbF_6_, DCM, 40 °C, 1 h; (iii) PPh^F^_3_AuCl, AgSbF_6_, DCM, 40 °C, 4 h.

In 2020 we reported a very efficient method to prepare benzo‐fused BODIPYs via Au^I^‐catalyzed cycloisomerization.^[17]^ They displayed strong, red‐shifted absorptions that mainly arise from LUMO stabilization. This led us to envision that stable anthracene‐fused oligo‐BODIPYs with different lengths and geometries may be accessed by the cyclization of key precursors **1**–**4** (Scheme 1). Herein, we report the synthesis, characterization, and electronic properties of a series of fully π‐conjugated anthracene‐fused BODIPYs dimers and trimers (**DIM‐para/meta** and **TRIM‐para/meta**; Figure 1).

## Results and Discussion

First, key precursors **1** and **2** were synthesized by Pd‐catalyzed cross coupling between **A** and **C/D**, respectively (Scheme 1). The number of BODIPY equivalents in this Suzuki coupling allowed the synthesis of key intermediates **I1** and **I2**, which were later employed to assemble precursors **3** and **4** by cross‐coupling with **B**. Then, the cycloisomerization of **1** and **2** catalyzed by the in situ generated PPh_3_^F^Au^+^ complex led to **DIM‐para** and **DIM‐meta** in 93 % and 91 % yields, respectively. Similarly, the cyclo‐isomerization of **3**–**4** furnished **TRIM‐para** and **TRIM‐meta** in 67 % and 72 % yields, respectively.

**Figure 2:**
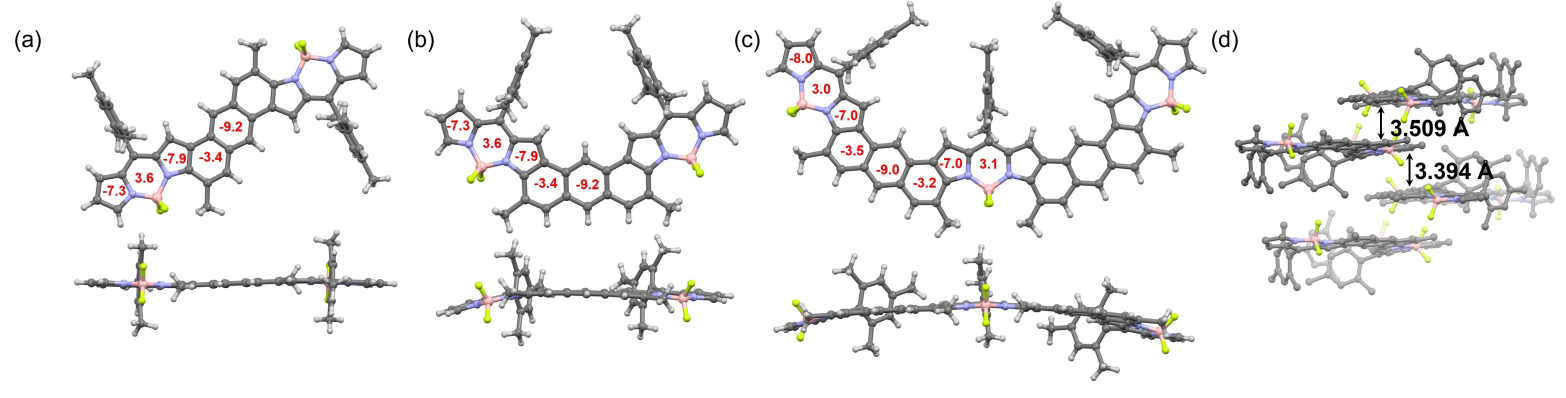
Front and side‐views of the X‐ray structure of a) **DIM‐para**, b) **DIM‐meta**, and c) **TRIM‐meta**. d) solid‐state packing of **TRIM‐meta**. The red numbers within the six‐ and five‐membered rings indicate the NICS(0) values calculated at the B3LYP/6‐31+G(d,p) level.

Single crystals of **DIM‐para**, **DIM‐meta**, and **TRIM‐meta** suitable for X‐ray diffraction analyses were grown by slow diffusion of hexane into their chloroform solution.^[18]^ The resolved structures and selected crystallographic data are summarized in Figure 2 and Table 1. As shown in the side‐view images, **DIM‐para** displays the expected planar π‐backbone, while **DIM‐meta** and **TRIM‐meta** exhibit a slightly curved structure, probably due to the steric repulsion between the mesityl groups. Remarkably, C−C bond length analyses revealed that the distortion of anthracene correlates with size of the π‐system, that is, either dimers or trimers, but is independent of the position to which BODIPY is fused. Thus, most of the C−C lengths in **DIM‐para** and **DIM‐meta** are similar to those of anthracene, whereas **TRIM‐meta** exhibits a wider C−C bond length distribution. Importantly, in all cases the *b* bonds (ring A) are in the range of 1.36–1.39 Å, which is significantly shorter than the remaining C−C bonds, and which is close to that of typical olefins (1.35 Å). Additionally, the C−C bonds of the *B* rings (d, e and e′) show bond lengths similar to that of a benzene ring (1.37–1.44 Å). All of these structural findings point to an effective π‐conjugation, in which the aromatic character of the anthracenes is mainly localized in the central rings. Such a conclusion nicely agrees with the calculated NICS(0) values (red numbers, Figure 2a–c), which reveal a lower aromatic character in the benzenic rings directly fused to BODIPY. A similar trend is also observed in the DFT predicted structure of **TRIM‐para** (See Supporting Information). Interestingly, the π‐system size also determines the solid‐state organization (Figure 2d and S3.1). In the case of **DIM‐para** and **DIM‐meta**, dipole‐dipole interactions lead to a disordered packing with no orientational preferences rather than π‐π stacking. This is in accordance with our previous work, where we observed that the aromatic ring fusion at both pyrroles is needed to knit a directional solid state π‐network. In stark contrast, close π‐π contacts with distances of 3.50 and 3.94 Å are found in the crystal packing of **TRIM‐meta** (Figure 2d). These interactions enable the formation of a highly directional packing, where the molecules are parallelly oriented.

**Table 1:** Selected C−C bond lengths of BODIPY‐anthracene conjugates in comparison with anthracene.

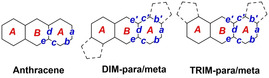					
	*a* [Å]	*b*/*b′* [Å]	*c*/*c′* [Å]	*d* [Å]	*e*/*e′* [Å]
Anthracene	1.42	1.37	1.43	1.43	1.40
**DIM‐meta/para**	1.43	1.36/1.43	1.43	1.42	1.39–1.40
**TRIM‐meta/para**	−1.23	1.38–1.39 /1.43	1.40–1.44 /1.45	1.4–1.44	1.39–1.45

**Figure 3:**
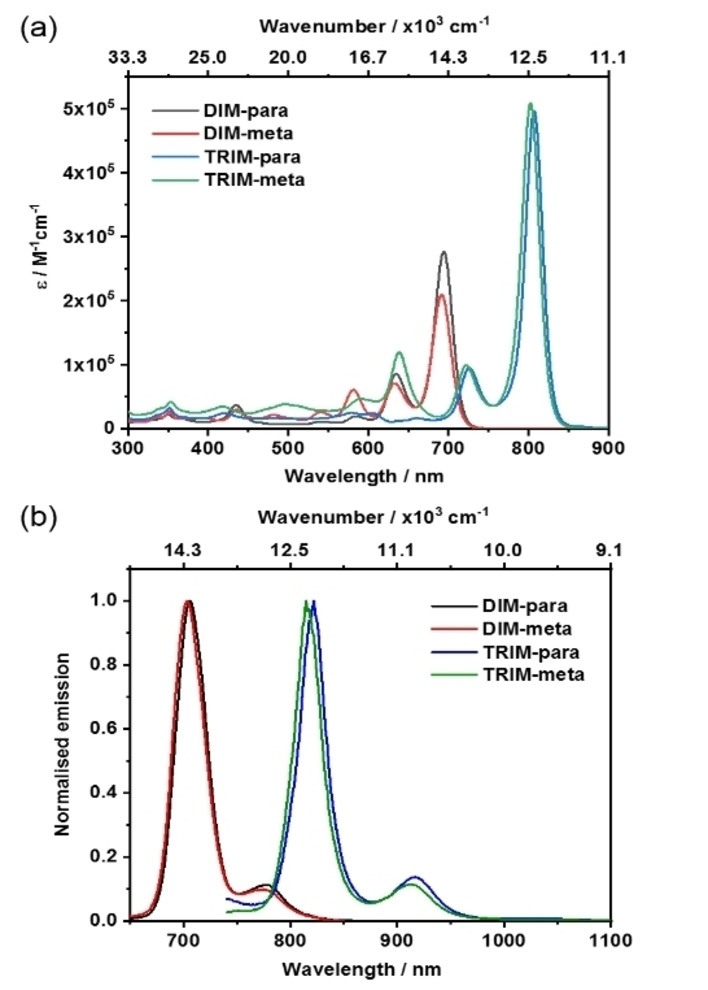
a) Absorption spectra of **DIM‐para**, **DIM‐meta**, **TRIM‐para** and **TRIM‐meta** recorded in toluene at room temperature. b) Normalized steady‐state fluorescence spectra of **DIM‐para**, **DIM‐meta** (*λ*_ex_—630 nm), **TRIM‐para** and **TRIM‐meta** (*λ*_ex_—720 nm) recorded in toluene at room temperature.

The photophysical properties of BODIPY‐anthracene oligomers were firstly characterized by steady‐state UV/Vis absorption (Figure 3). Compared to the absorption features of precursors **1**–**4** and previously reported benzene‐fused homologues (Figure S4.1), all oligo‐BODIPY exhibit bathochromically shifted absorptions, indicating an effective π‐conjugation after the cycloisomerization. These absorptions are mainly characterized by S_0_ → S_1_ transitions. In the case of **DIM‐para** and **DIM‐meta**, such a transition is centered at around 690 nm. Notably, the S_0_→S_1_ transition is 100 nm red‐shifted in the case of **TRIM‐para** and **TRIM‐meta**. Our findings suggest that the conjugation remains well‐extended over the entire π‐framework upon coupling of additional BODIPY‐anthracene units. Accordingly, the extinction coefficient increases by two when moving from the dimers to the trimers, reaching a remarkable 500 000 M^−1^ cm^−1^ (Table S6.1). Importantly, the absorptions of all BODIPY‐anthracene systems were concentration independent, indicating the absence of any intermolecular aggregation.

**Figure 4:**
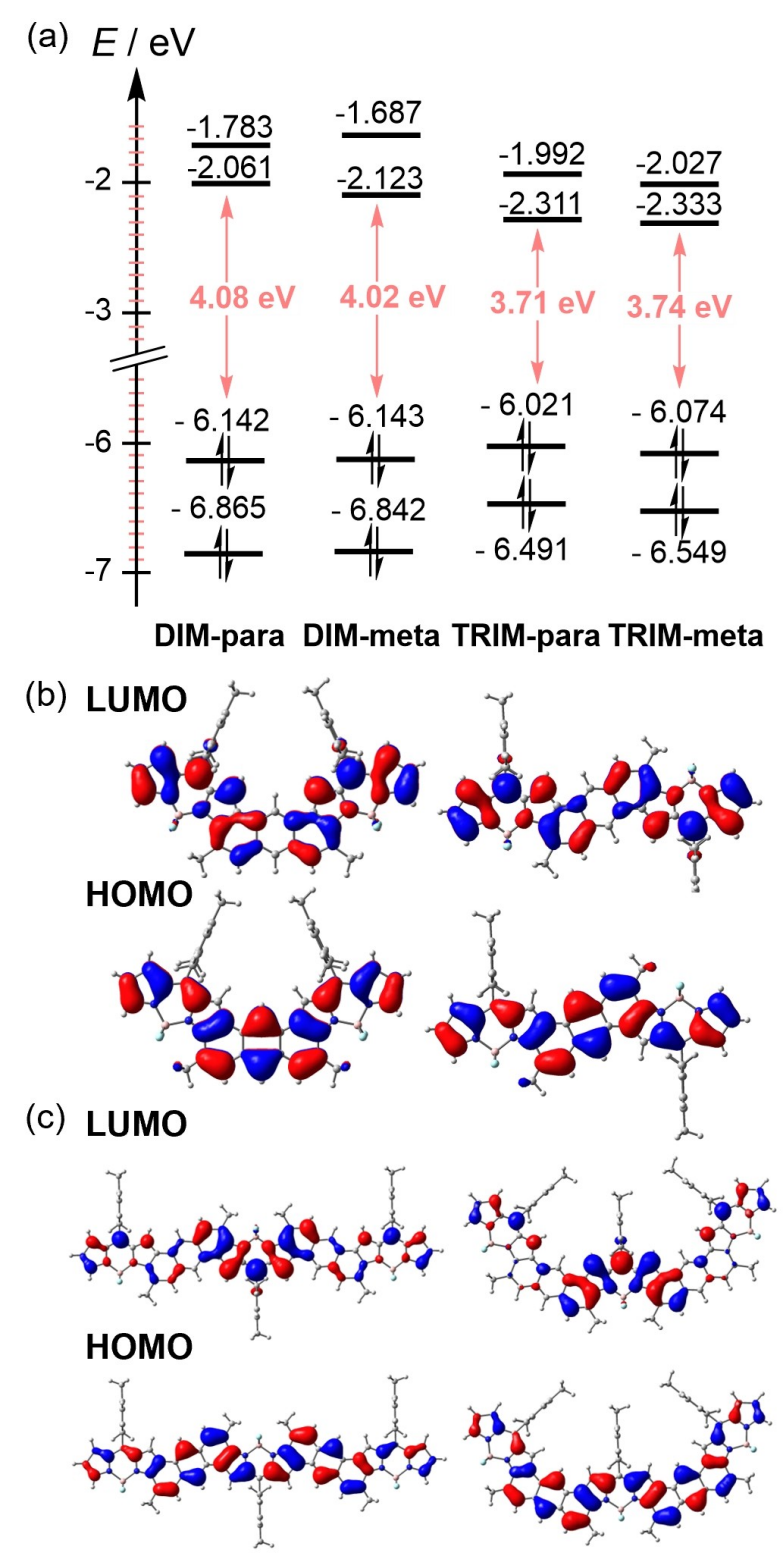
a) Energy values and Kohn–Sham orbital representations of the HOMO and LUMO of b) **DIM‐para**/**meta** and c) **TRIM‐para**/**meta**. All calculations were performed at the CAM‐B3LYP/6‐31G level.

Further insights into the optical properties and electronic structure of BODIPY‐anthracene oligomers were gathered by means of DFT calculations (CAM−B3LYP/6‐31G(d,p) level; See Supporting Information for further details). As shown in Figure 4, the HOMO and LUMO of **DIM‐para** and **DIM‐meta** are well delocalized over the anthracene and BODIPY units. On the contrary, the HOMO–LUMO orbital representation of **TRIM‐para** and **TRIM‐meta** revealed that, while the HOMO is extended over the whole system, the LUMO is mainly located at the central BODIPY. This finding is rationalized by considering previous work by Wakamiya et al.^[14b]^ They demonstrated that the benzo‐fusion at the pyrrole significantly improves the electron accepting features of BODIPYs. To evaluate whether such a stronger electron acceptor character of the central BODIPY polarizes the π‐cloud, the electrostatic potential surface was simulated by DFT (Figure S5.2). The resulting maps showed that the electron density is equally distributed in the dimers and trimers. It is also important to highlight that the lower band‐gap found in **TRIM‐para** and **‐meta** compared to **DIM‐para** and **‐meta** mainly arises from the LUMO stabilization. This explains their stability (See energy values of Figure 4). Next, time‐dependent (TD) DFT were carried out to assign the nature of the UV/Vis absorptions. Selected TD‐DFT transition energies, oscillator strengths (f) and molecular orbital configurations are shown in Table S5.1. In all cases, the S_0_→S_1_ transitions mostly correspond to the HOMO–LUMO transitions. Moreover, **DIM‐meta** and **TRIM‐meta** display additional weak shoulders at lower wavelengths, which arise from a combination of multiple HOMO‐*n* → LUMO+*m* (*n*,*m*=0–2) transitions, and, which are in good agreement with the experimental results. Taking the HOMO–LUMO representations of **TRIM‐para** and **TRIM‐meta** into account, the S_0_→S_1_ transitions are based on a charge‐transfer character, where the electronic density flows from the outer BODIPY‐anthracene to the central BODIPY. This push‐pull character might also be the basis for the substantial red‐shifts observed in the trimers in comparison to the dimers. It should, however, be noted that the transferred charges are delocalized over several atoms (i.e., central BODIPY unit). From this we expect a strong solvatochromism. To confirm this hypothesis, the absorption spectra of BODIPY‐anthracene systems were recorded in different solvents such as THF and DCM, and solvent polarity‐dependent blue shifts could be observed in all the samples (Figure S6.1 and Table S6.1).

The oligomers exhibited vibrationally resolved fluorescence spectra, with their maxima Stokes‐shifted by around 15–20 nm (Figure 3). **DIM‐para** and **DIM‐meta** in toluene revealed maxima centered at 705 nm and shoulders at around 775 nm. In the case of **TRIM‐para** and **TRIM‐meta**, the red‐shifted fluorescence spanned the spectral range of 750 to 1000 nm, with maxima at ≈810 nm and shoulders at ≈900 nm. The quantum yields of **DIM‐para** and **DIM‐meta** in toluene were determined to be 56 % and 59 %, respectively. Compared to the **DIM**‐oligomers, the **TRIM** counterparts exhibited reduced quantum yields of around 20–26 %, which is slightly higher than the reported quantum yields of benzene‐fused BODIPY trimers. Significant reduction in the quantum yields were also observed upon changing the solvent from non‐polar toluene to polar THF and DCM, suggesting the possibility of non‐radiative deactivation pathways. Furthermore, fluorescence lifetimes were determined using time‐correlated single photon counting (TCSPC) (Table S6.1). All decays were monoexponential, with lifetimes ranging from 1.8 to 3.3 ns in toluene, THF and DCM. A minor reduction in lifetime was observable upon increasing the solvent polarity.

**Figure 5:**
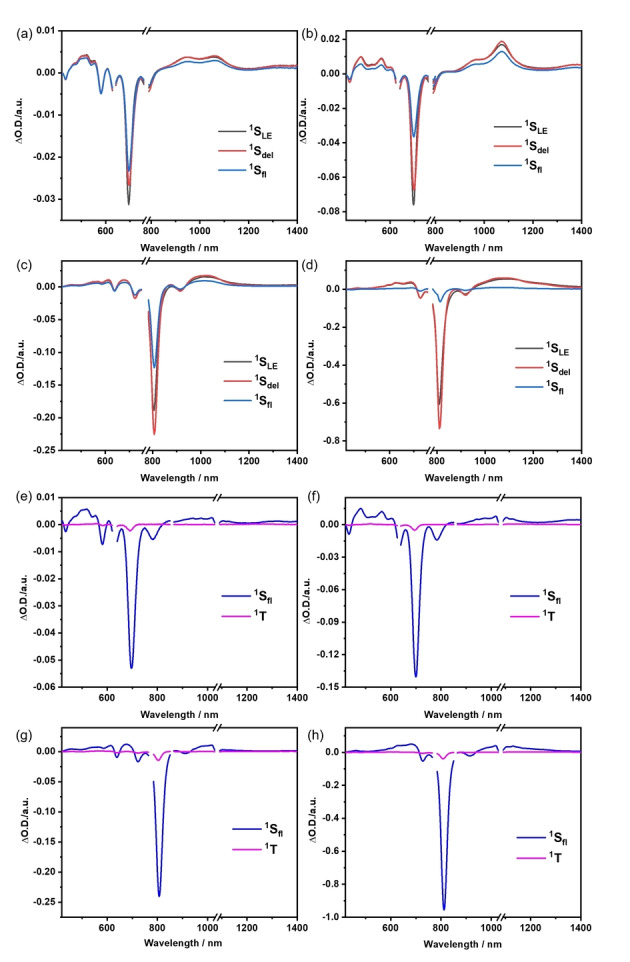
Evolution associated spectra reconstructed from the sequential global analysis of fs‐TA spectra of a) **DIM‐meta**, b) **DIM‐para**, c) **TRIM‐meta** and d) **TRIM‐para** and of ns‐TA spectra of e) **DIM‐meta**, f) **DIM‐para**, g) **TRIM‐meta** and h) **TRIM‐para** in toluene.

To investigate the excited state decay dynamics of the BODIPY‐anthracene oligomers in toluene, femtosecond (fs‐TA) as well as nanosecond transient absorption (ns‐TA) pump‐probe measurements were carried out. Global sequential analysis of the TA spectra was performed using the GloTarAn^[19]^ program by employing kinetic models with four species. The evolution associated spectra (EAS) deconvoluted from the fs‐TA and ns‐TA spectra of the oligomers are as shown in Figure 5. Upon 630 nm photoexcitation of **DIM‐meta** and **DIM‐para**, an intense ground state bleaching at 695 nm along with singlet excited state absorptions in the 450–550 nm and 900–1100 nm regions, were observed. The first species, with its 4 ps lifetime, is assigned to be the local singlet excited state (^1^S_LE_). It undergoes relaxation to populate the second species, which also exhibits singlet excited state characteristics. We postulate that this species corresponds to the delocalized singlet excited state (^1^S_del_). It lives for 279 and 800 ps in **DIM‐meta** and **DIM‐para**, respectively, which suggests a more effective electron delocalization in the planar π‐backbone of **DIM‐para** compared to **DIM‐meta**. Considering that the TCSPC measurements lack any evidence for its existence, we conclude that it is subject to a non‐radiative decay. Further relaxation of the delocalized state results in the formation of the third species, which is the fluorescent singlet excited state (^1^S_fl_). It decays with 3.11 and 3.06 ns in **DIM‐meta** and **DIM‐para**, respectively, which is in agreement with the lifetime values obtained from TCSPC. From the ns‐TA spectra, a subtle contribution of intersystem crossing is derived from the overall decrease in ground state bleaching. The subsequently formed triplet excited state (^1^T) is characterized by absorption maxima at around 500 nm and in the 1100–1300 nm range. From there, ground state recovery takes place with 163.57 and 180.89 ns in **DIM‐meta** and **DIM‐para**, respectively. In the case of **TRIM‐meta** and **TRIM‐para**, 775 nm photoexcitation leads to the same excited state deactivation mechanism seen for **DIM‐meta** and **DIM‐para**. In particular, the initially formed local singlet excited states, characterized by ground state bleaching at 810 nm and excited state absorptions at 500–700 nm and in the IR region, feature lifetimes of 10 and 8 ps for **TRIM‐meta** and **TRIM‐para**, respectively. Next, the non‐radiative delocalized singlet excited state evolves. 555 ps and 1.15 ns are the lifetimes for **TRIM‐meta** and **TRIM‐para**. Relative to **DIM‐meta** and **DIM‐para**, such a substantial increase in lifetime reflects the electronic communication in **TRIM‐meta** and **TRIM‐para**, which is based on an extended π‐conjugation, as seen, for example, in the steady‐state absorption spectra. It is also interesting to note that the lifetimes of the second species in **DIM‐para** and **TRIM‐para** are significantly longer than those in **DIM‐meta** and **TRIM‐meta**. This suggests that the communication between the central BODIPY and the peripheral BODIPY‐anthracenes is much more efficient in the planar π‐backbone of **DIM‐para** and **TRIM‐para**. The third species is the fluorescent singlet excited state. The lifetimes are 2.46 ns in **TRIM‐meta** and 2.00 ns in **TRIM‐para**. Finally, the triplet excited state is formed as a minor component, which repopulates the ground state with a time delay of 99.78 and 66.36 ns in **TRIM‐meta** and **TRIM‐para**, respectively.

To experimentally assess the frontier molecular orbitals of **DIM‐para**, **DIM‐meta**, **TRIM‐para** and **TRIM‐meta**, their oxidations and reductions were determined in dichloromethane by cyclic voltammetry using 0.1 M TBAPF_6_ as electrolyte and Fc/Fc^+^ as the internal reference (Table 2 and Figure S8.1). Remarkably, all of them displayed a similar redox behavior, by presenting one reversible oxidation and two reversible reductions. Regardless of the size of the π‐system, the oxidation is centered at around +0.65 V. On the other hand, the first reduction of **DIM‐para** and **DIM‐meta** were observed at around −1.11 and −1.14 V, respectively, and that of **TRIM‐para** and **TRIM‐meta** were at around −0.98 and −0.99 V, respectively, suggesting that the elongation of the BODIPY‐anthracene system improves the accepting properties of the material. This implies a lowering of the band gap, which is in line with the spectroscopic results. The second reduction is similar in the four systems, being slightly lower in the case of **DIM‐meta** and **TRIM‐meta**. Considering the energy level of Fc/Fc^+^ with respect to the vacuum level (−4.8 eV), the HOMO and LUMO energy levels of the BODIPY‐anthracene oligomers can be estimated using its experimental redox potentials. As shown in Table 2, the **TRIM**‐oligomers exhibit a LUMO at ≈−3.8 V, which is comparable to other electron‐poor acceptors, such as PC_60_BM (−3.91 V). Therefore, such an acceptor character, together with their excellent photoresponse in the NIR range, render **TRIM‐para** and **TRIM‐meta** promising candidates for (opto)electronic applications.

**Table 2:** Electrochemical oxidation and reduction of **DIM‐para**, **DIM‐meta**, **TRIM‐para**, and **TRIM‐meta** obtained by cyclic voltammetry in dichloromethane.^[a]^

	*E* ^Red^_2_ [V]	*E* ^Red^_1_ [V]	*E* ^Ox^_1_ [V]	HOMO [eV]	LUMO [eV]
**DIM‐para**	−1.23	−1.11	0.67	−5.47	−3.69
**DIM‐meta**	−1.26	−1.14	0.65	−5.45	−3.66
**TRIM‐para**	−1.23	−0.98	0.67	−5.47	−3.82
**TRIM‐meta**	−1.27	−0.99	0.66	−5.46	−3.81

[a] 0.1 M TBAPF_6_ was used as electrolyte and Fc/Fc^+^ as internal reference.

## Conclusion

In summary, we have synthesized large π‐conjugated systems comprising either three (**DIM‐para** and **‐meta**) or five (**TRIM‐para** and **‐meta**) alternating BODIPY‐anthracene units, which exhibit intriguing properties that are size and symmetry‐dependent. Concerning structural features, larger sizes of the π‐conjugated systems result in a stronger distortion of the anthracene units. With respect to supramolecular features, the larger π‐systems as in **TRIM‐meta** relative to **DIM‐para** and **‐meta** foster π‐π interactions and yield face‐to‐face supramolecular organizations in the solid‐state. In the context of optical features, all π‐conjugated systems exhibit absorption spectra that are dominated by S_0_→S_1_ transitions. Notable, the long wavelength absorption and fluorescence of **TRIM‐para** and **TRIM‐meta** is subject to a remarkable red‐shifted in comparison to **DIM‐para** and **DIM‐meta** and, in turn, reach well into the NIR region. By means of time‐resolved pump‐probe experiments, a more efficient π‐conjugation in **TRIM‐meta** and **TRIM‐para** was corroborated when compared to **DIM‐meta** and **DIM‐para**. Finally, we found that the redox properties are also affected by the length of the π‐system as **TRIM‐para** and **TRIM‐meta** are stronger electron acceptors than **DIM‐para** and **DIM‐meta**.

Given the intriguing properties of our BODIPY‐anthracene conjugates, we conclude that BODIPYs are excellent building blocks for the preparation of highly π‐conjugated platforms. Current efforts towards even larger systems with different geometries and dyes are ongoing in our laboratories.

## Conflict of interest

The authors declare no conflict of interest.

1

## Supporting information

As a service to our authors and readers, this journal provides supporting information supplied by the authors. Such materials are peer reviewed and may be re‐organized for online delivery, but are not copy‐edited or typeset. Technical support issues arising from supporting information (other than missing files) should be addressed to the authors.

Supporting Information

Supporting Information

## Data Availability

The data that support the findings of this study are available from the corresponding author upon reasonable request.
