# Supplementary material for: Anthracene‐Fused Oligo‐BODIPYs: A New Class of π‐Extended NIR‐Absorbing Materials
Source: Angew Chem Int Ed Engl. 2022 Dec 8;62(5):e202214543. doi: 10.1002/anie.202214543 (PMC10107270; doi:10.1002/anie.202214543)
Supplement: Supplementary file 1 — Supporting Information [file ANIE-62-0-s001.pdf]

Angewandte Chemie
Eine Zeitschrift der Gesellschaft Deutscher Chemiker

Supporting Information

Anthracene-Fused Oligo-BODIPYs: A New Class of π-Extended NIR-Absorbing Materials

J. Labella, G. Durán-Sampedro, S. Krishna, M. V. Martínez-Díaz, D. M. Guldi, T. Torres**

Supporting Information

<u>Table of content</u>

1. Instrumentation and materials S2
2. Synthetic Procedures and Compound Data S3

 Synthesis and characterization of boronic ester **B**........ S3

 Synthesis and characterization of BODIPYs **1-4** and **I1-2**........ S4

 Synthesis and characterization of BODIPY-anthracene oligomers........ S7

 Crystal packing of BODIPYs **DIM-para and DIM-meta**. S9

3. Absorption and Fluorescence Spectra of 1-4 and I1-2 .... S12
4. NMR spectra........ S13
5. Computational Studies S30

 Theorical structure and selected C-C bond lengths of **TRIM-para** S30

 Electrostatic potential surfaces of BODIPY-anthracene oligomers S30

 Frontier molecular orbitals of BODIPY-anthracene oligomers S31

 TD-DFT calculations........ S31

6. Photophysical characterization........ S32
7. Transient absorption studies........ S35
8. Electrochemistry........ S36
9. References S37

1. **Instrumentation and materials**

Nuclear magnetic resonance spectra (^{1}H-, ^{13}C-, ^{19}F-NMR) were recorded on a Bruker AV-300 or a Bruker DRX-500 spectrometers either in the Organic Chemistry Department or in the Interdepartmental Investigation Service of UAM. Deuterated solvent employed in each case is indicated in brackets, and its residual peak was used to calibrate the spectra using literature reference δ ppm values.[1] All the experiments were recorded at room temperature.

Mass spectra (MS) and high-resolution mass spectra (HRMS) were recorded in the Interdepartmental Investigation Service of UAM, employing Electronic Impact (EI), or Matrix-Assisted Laser Desorption/Ionization Time-Of-Flight (MALDI-TOF), using a VG-AutoSpec spectrometer for EI, and a Bruker-Ultraflex-III spectrometer, with a Nd:YAG laser operating at 355 nm, for MALDI-TOF. The matrixes and internal references employed are indicated for each spectrum. Infrared Spectra were recorder in solid state on a Bruker Vector 22 spectrophotometer.

Ultraviolet and visible (UV-Vis) spectra were recorded using solvents in the spectroscopic grade in the Organic Chemistry Department of UAM employing a JASCO-V660 spectrophotometer, and PerkinElmer Lambda 2 dual beam absorption spectrophotometer. The logarithm of the molar extinction coefficient (ε) is indicated in brackets for each maximum. Likewise, fluorescence measurements were carried out with a JASCO-V8600 spectrofluorometer and Horiba Jobin Yvon FluoroMax-3 emission spectrometer. NIR emission spectra were recorded on Horiba Jobin Yvon FluoroLog3 spectrometer using 450 W xenon lamp and Symphony InGaAs array detector in combination with an iHR320 imaging spectromter.

Fluorescence quantum yields (ϕ_F) of **1-4** and **I1-2** were determined in DCM, and that of BODIPY-anthracene oligomers were measured in toluene, THF and DCM, and calculated using the following equation:[2]

$$\phi_F^S = \phi_F^R \left(\frac{\mathrm{Grad_S}}{\mathrm{Grad_R}}\right)\left(\frac{\eta_S}{\eta_R}\right)^2$$

Scripts R and S indicate reference and sample, respectively. Grad is the gradient from the plot of the integrated fluorescence intensity versus the absorption and η is the refractive index of the solvent (the excitation wavelength and the reference compounds are indicated in each case). For **1-2** and **I1-2** rhodamine 6G was used as the standard (Φ_F = 0.95 in ethanol).[3] For **3-4** zinc phthalocyanine was used as the standard (Φ_F = 0.45 in PrOH).[3]

Single-crystal X-ray diffraction data collection for structure determinations were collected in the Interdepartmental Investigation Service [4] of UAM at Bruker KAPPA APEX II CCD area-detector X-ray diffractometer operating with graphite monochromated and Mo Kalpha radiation (λ = 0.71073 Å). The data are absorption corrected with the program SADABS. Intensities are calculated with the SAINT software, which also incorporates polarization and Lorentz effect corrections. The structures were solved and refined using the Bruker SHELXTL Software Package.

Femtosecond and nanosecond transient absorption measurements were performed using HELIOS (0 to 5000 ps) and EOS (1 ns to 350 μs) pump/probe setup from Ultrafast Systems. CPA2101 and 2110 Ti:Sapphire amplifier (775 nm output, 1 kHz repetition rate, 150 fs pulse width) from Clark-MXR Inc. was used as the laser source. Excitation pulse of 630 nm was generated using a NOPA. The white light for femtosecond experiments was generated by focusing the 775 nm fundamental output to a Sapphire crystal. The white light for nanosecond transient measurements was generated from a supercontinuum laser source with 2 kHz repetition rate and 1 ns pulse width. Samples were taken in 2 × 10 mm optical

glass cuvettes and were purged with argon for 20 mins. Global sequential analysis of the transient absorption data was performed using GloTarAn software.[5]

Electrochemical characterizations were performed with a three-electrode setup on AutoLab PGStat 30 instrument. The measurements were carried out in dichloromethane using 0.1 M tetrabutylammonium hexafluorophosphate ($TBAPF_6$) as electrolyte and compound concentration of approx. 10^{-4} M. A platinum rod was used as working electrode, platinum wire served as counter electrode and Ag/AgCl as reference electrode. Ferrocene (Fc) was used as the internal standard and all the potentials were noted relative to the Fc/Fc+ couple. Scan rate was 100 mV s^{-1}. The data were recorded with NOVA 2.0 software.

The monitoring of the reactions has been carried out by thin layer chromatography (TLC), employing aluminium sheets coated with silica gel type 60 F254 (0.2 mm thick, Merck). The analysis of the TLCs was carried out with an UV lamp of 254 and 365 nm. Purification and separation of the synthesized products was performed by normal-phase column chromatography, using silica gel (230-400 mesh, 0.040-0.063 mm, Merck). Eluents along with the relative ratio in the case of solvent mixtures are indicated for each particular case.

Chemicals were purchased from commercial suppliers and used without further purification. Dry solvents were purchased from commercial suppliers in anhydrous grade or thoroughly dried before use employing standard methods. Solid, hygroscopic reagents were dried in a vacuum oven before use.

The synthesis and characterization **2,6-Br$_2$BDP**, **A**, 1,4-dibromo-2,5-di(prop-1-ynyl)benzene (**C**) and 1,5-dibromo-2,4-di(prop-1-ynyl)benzene (**D**) have been previously reported.[6]

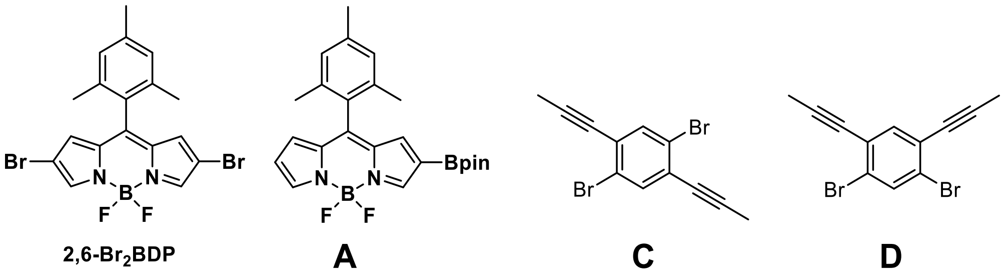

2. **Synthetic Procedures and Compound Data**

<u>Synthesis and characterization of boronic ester **B**</u>

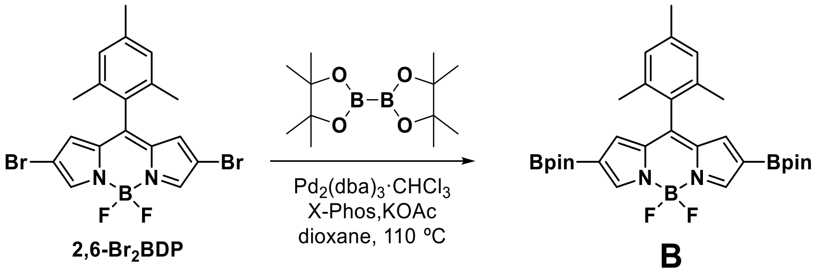

Scheme S2.1. Synthesis of building block **B**.

A Schlenk tube was charged with **2,6-Br$_2$BDP** (200 mg, 0.43 mmol), $Pd_2(dba)_3$•$CHCl_3$ (8 mg, 9.0 µmol), X-Phos (16.4 mg, 34.4 µmol), bis(pinacolato)diboron (655 mg, 2.58 mmol), KOAc (254 mg, 2.58 mmol). The Schlenk tube was capped with a rubber septum and then evacuated and backfilled with N_2 sequence was carried out two times). 1,4-Dioxane (5 mL) was added via syringe through the septum,

and the Schlenk tube was sealed. The reaction mixture was heated to 110 °C until **2,6-Br$_2$BDP** had been completely consumed by TLC analysis. At this point the reaction mixture was allowed to cool to room temperature. The reaction solution was then filtered through a thin pad of Celite and eluted with CH_2Cl_2. After removal of solvents in vacuo, the mixture was purified by silica-gel short column chromatography (CH_2Cl_2 as eluent) and solvent was removed in vacuo. The residue was washed with hexane and recrystallization from CH_2Cl_2/hexane afforded **B** (193.4 mg, 0.34 mmol) in 80% yield as green crystals. **^{1}H-NMR** (500 MHz, $CDCl_3$): δ (ppm) = 8.22 (s, 2 H), 7.09 (s, 2 H), 6.90 (s, 2 H), 2.33 (s, 3 H), 2.06 (s, 6 H), 1.28 (s, 24 H).; **^{13}C-NMR** (75.50 MHz, $CDCl_3$): δ (ppm) = 151.0, 148.8, 139.0, 138.1, 137.0, 136.4, 129.7, 128.3, 83.9, 24.9, 21.2, 20.1.; **^{19}F NMR** (471 MHz, $CDCl_3$): δ (ppm) = -145.65 (m). **^{11}B-NMR** (160 MHz, $CDCl_3$).: δ (ppm) = 0.35 (t, J_{B-F} = 28.8 Hz).; **HRLSI-MS** (MADLDI-TOF): Calculated for $C_{30}H_{39}B_3F_2N_2O_4$; 562.3148, found, 562.3139, ppm error = 2.8; **UV/vis** ($CHCl_3$): λ_{max} (nm) (log ε) = 504 (4.3). **Φ_F** (exc. λ = 50 nm) = 0.52.; **FT-IR**: ν (cm^{-1}) = 3055, 3011, 2988, 1569, 1408, 1299, 1225, 1097, 1010, 779, 711.; **Mp** > 200 ºC

Synthesis and characterization of BODIPYs **1-4** and **I1-2**

General procedure A: Suzuki cross-coupling.

A Schlenk tube was charged with the appropriate BODIPY boronic acid (1-2 eq), ^{t}BuXPhos Pd G3 (0.2eq), aryl bromide (1-2 eq) and K_3PO_4 (2.2-4.4 eq) were dissolved in 5 ml of a previously degassed mixture of THF/water (2:3; v/v) under argon atmosphere. The reaction was stirred at room temperature for 10 min. After that, the crude of the reaction was extracted with CH_2Cl_2 (3x20 mL), dried over $MgSO_4$, filtered and concentrated. The residue was purified by column chromatography on silica gel using a suitable eluent, as specified in each case.

BODIPY **I1**

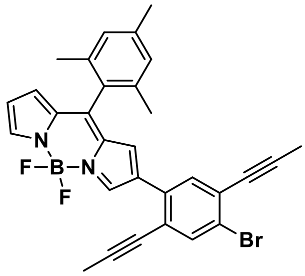

General procedure A was employed with the following quantities: **A** (100 mg, 0.23 mmol), **C** (71.5 mg, 0.23 mmol), ^{t}BuXPhos Pd G3 (36.5 mg, 46 μmol) and K_3PO_4 (107 mg, 0.51 mmol). The crude product was purified by column chromatography (0–20 % toluene in heptane) to afford **I1** (30 mg, 24%) as a red solid. **^{1}H-NMR** (500 MHz, $CDCl_3$): δ (ppm) = 8.44 (s, 1 H), 7.94 (s, 1 H), 7.62 (s, 1 H), 7.43 (s, 1 H), 7.02(s, 1 H), 6.97(s, 2 H), 6.72 (d, J = 4.1 Hz, 1H), 6.50 (d, J = 4.1 Hz, 1H), 2.38 (s, 3 H), 2.15 (s, 6 H), 2.11 (s, 3 H), 1.98 (s, 3 H).; **^{13}C-NMR** (126 MHz, $CDCl_3$): δ (ppm) = 147.6, 144.7, 143.7, 139.0, 136.6, 136.4, 135.7, 135.3, 133.2, 131.7, 131.4, 130.3, 129.7, 128.2, 127.1, 125.6, 123.1, 121.6, 118.8, 93.2, 92.5, 78.5, 78.2, 21.2, 20.1, 4.7, 4.4.; **^{19}F NMR** (471 MHz, $CDCl_3$): δ (ppm) = -145.74 (m). **^{11}B-NMR** (160 MHz, $CDCl_3$): δ (ppm) = 0.32 (t, J_{B-F} = 28.9 Hz). **HRLSI-MS** (MALDI-TOF): calculated for $C_{30}H_{24}BBrF_2N_2$: 541.2467, Found: 541.2478. ppm error = 3.0.; **UV/vis ($CHCl_3$)**: λ_{max} (nm) (log ε) = 537 (4.7). **Φ_F** (exc. λ = 510 nm) = 0.44.; **FT-IR**: ν (cm^{-1}) = 3075, 3069, 3030, 3019, 2910, 2890, 1517, 1376, 1228, 1096, 972, 799.; **Mp** > 200 ºC

BODIPY **1**

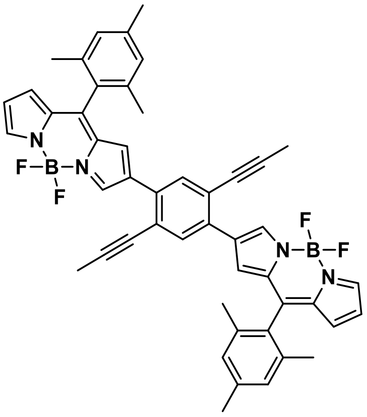

General procedure A was employed with the following quantities: **A** (100 mg, 0.23 mmol), **C** (30.5 mg, 0.11 mmol), ^{t}BuXPhos Pd G3 (36.5 mg, 15.5 μmol) and K_3PO_4 (107 mg, 0.51 mmol). The crude product was purified by column chromatography (0–20 % toluene in heptane) to afford **1** (70 mg, 82%) as a red solid. **^{1}H-NMR** (500 MHz, $CDCl_3$): δ (ppm) = 8.50 (s, 2H), 7.92 (s, 2H), 7.47 (s, 2H), 7.06 (s, 2H), 6.97 (s, 4H), 6.70 (d, *J* = 4.1 Hz, 2H), 6.49 (d, *J* = 4.1 Hz, 2H), 2.38 (s, 6H), 2.15 (s, 12H), 1.98 (s, 6H).; **^{13}C-NMR** (126 MHz, $CDCl_3$): δ (ppm) = 147.3, 144.3, 144.3, 139.0, 136.4, 135.6, 135.4, 132.8, 132.2, 131.9, 130.0, 129.7, 128.2, 127.0, 120.7, 118.6, 92.3, 79.4, 21.2, 20.1, 4.4.; **^{19}F NMR** (471 MHz, $CDCl_3$): δ (ppm) = -145.75 (m); **^{11}B-NMR** (160 MHz, $CDCl_3$): δ (ppm) = 0.33 (t, $J_{B\text{-}F}$ = 28.9 Hz).; **HRLSI-MS** (MALDI-TOF): calculated for: $C_{48}H_{40}B_2F_4N_4$: 770.3375, Found: 770.3383. ppm error = 2.4.; **UV/vis ($CHCl_3$)**: λ_{max} (nm) (log ε) = 563 (4.1). **Φ_F** (exc. λ = 510 nm) = 0.42.; **FT-IR**: ν (cm^{-1}) = 3073, 3012, 2977, 2860, 1561, 1419, 1396, 1299, 1226, 1097, 1012, 779, 707.; **Mp** > 200 °C

BODIPY **I2**

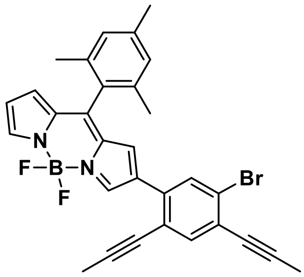

General procedure A was employed with the following quantities: **A** (100 mg, 0.23 mmol), **D** (71.5 mg, 0.23 mmol), ^{t}BuXPhos Pd G3 (36.5 mg, 46 μmol) and K_3PO_4 (107 mg, 0.51 mmol). The crude product was purified by column chromatography (0–20 % toluene in heptane) to afford **1c** (45 mg, 36%) as a red solid . **^{1}H-NMR** (500 MHz, $CDCl_3$): δ (ppm) = 8.49 (s, 1H), 7.98 (s, 1H), 7.66 (s, 1H), 7.48 (s, 1H), 7.06 (s, 1H), 7.02 (s, 2H), 6.76 (d, *J* = 4.1 Hz, 1H), 6.55-6.54 (m, 1H), 2.42 (s, 3H), 2.19 (s,6), 2.16 (s, 3H), 2.03 (s, 3H).; **^{13}C-NMR** (126 MHz, $CDCl_3$): δ (ppm) = 147.6, 144.7, 143.7, 139.0, 136.6, 136.4, 135.7, 135.3, 133.2, 131.7, 130.3, 129.7, 128.2, 127.1, 125.6, 123.1, 121.6, 118.8, 93.2, 92.5, 78.5, 78.2, 21.2, 20.1, 4.6, 4.4. ; **^{19}F NMR** (471 MHz, $CDCl_3$): δ (ppm) = -145.77 (m). **^{11}B-NMR** (160 MHz, $CDCl_3$): δ (ppm) = = 0.29 (t, *J* = 30.0 Hz). **HRLSI-MS** (MALDI-TOF): calculated for $C_{30}H_{24}BBrF_2N_2$: 541.2467, Found: 541.2472. ppm error = 2.7; **UV/vis ($CHCl_3$)**: λ_{max} (nm) (log ε) = 535 (4.4). **Φ_F** (exc. λ = 510 nm) = 0.38.; **FT-IR**: ν (cm^{-1}) = 3088, 3077, 3025, 2915, 2879, 1512, 1367, 1211, 1087, 972, 789.; **Mp** > 200 °C

BODIPY **2**

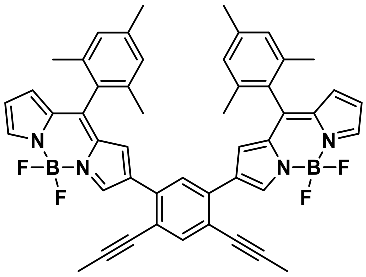

General procedure A was employed with the following quantities: **A** (100 mg, 0.23 mmol), **D** (30.5 mg, 0.11 mmol), ${}^tBuXPhos$ Pd G3 (36.5 mg, 15.5 μmol) and K_3PO_4 (107 mg, 0.51 mmol). The crude product was purified by column chromatography (0–20 % toluene in heptane) to afford **1d** (65 mg, 76 %) as a red solid. **^{1}H-NMR** (500 MHz, $CDCl_3$): δ (ppm) = 8.43 (s, 2H), 7.93 (s, 2H), 7.50 (s, 1H), 7.39 (s, 1H), 7.09 (s, 2H), 6.98 (s, 4H), 6.71 (d, *J* = 4.0 Hz, 2H), 6.49 (d, *J* = 4.1 Hz, 2H), 2.37 (s, 6H), 2.16 (s, 12H), 1.95 (s, 6H); **^{13}C-NMR** (126 MHz, $CDCl_3$): δ (ppm) = 147.6, 144.4, 144.1, 139.0, 138.3, 136.4, 135.7, 135.4, 133.7, 132.2, 130.1, 129.7, 128.3, 127.3, 126.0, 120.1, 118.6, 91.6, 79.0, 21.2, 20.1, 4.3. **^{19}F NMR** (471 MHz, $CDCl_3$): δ (ppm) = -145.68 (m); **^{11}B-NMR** (160 MHz, $CDCl_3$): δ (ppm) = 0.33 (t, *J* = 28.9 Hz); **HRLSI-MS** (MALDI-TOF): calculated for: $C_{48}H_{40}B_2F_4N_4$: 770.3375, Found: 770.3386, ppm error = 1.4.; **UV/vis ($CHCl_3$)**: λ_{max} (nm) (log ε) = 551 (4.5). **Φ_F** (exc. λ = 510 nm) = 0.37.; **FT-IR**: ν (cm^{-1}) =3063, 2897, 1550, 1413, 1135, 1107, 1092, 798. **Mp** > 200 °C.

BODIPY **3**

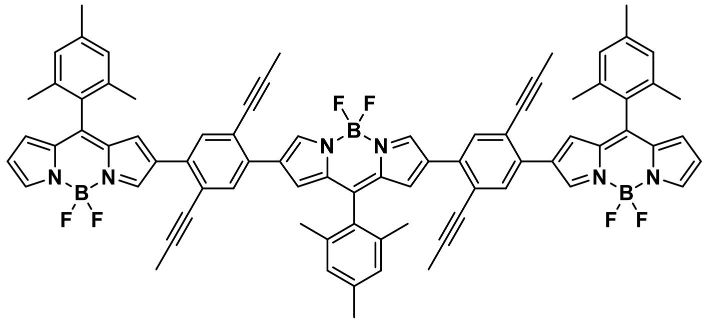

General procedure A was employed with the following quantities: **I1** (20 mg, 37 μmol), **B** (10 mg, 17 μmol), ${}^tBuXPhos$ Pd G3 (3 mg, 3.4 μmol) and K_3PO_4 (16 mg, 75 μmol). The crude product was purified by column chromatography (0–5 % ethyl acetate in toluene) to afford **3** (5.7 mg, 27%) as a blue solid. **^{1}H-NMR** (500 MHz, $CDCl_3$): δ (ppm) = 8.51 (s, 2H), 8.50 (s, 2H), 7.92 (s, 2H), 7.48 (s, 4H), 7.07 (s, 2H), 7.06 (s, 2H), 6.99 (s, 2H), 6.97 (s, 4H), 6.70 (d, *J* = 4.0 Hz, 2H), 6.49 (d, *J* = 4.1 Hz, 2H), 2.39 (s, 3H), 2.38 (s, 6H), 2.20 (s, 6H), 2.15 (s, 12H), 1.99 (s, 6H), 1.98 (s, 6H); **^{13}C-NMR** (126 MHz, $CDCl_3$): δ (ppm) = 147.3, 146.8, 144.3, 144.2, 139.1, 139.0, 136.5, 136.4, 135.6, 135.4, 132.8, 132.7, 132.2, 131.9, 130.0, 129.7, 128.2, 127.0, 126.8, 120.7, 118.6, 92.3, 92.3, 79.4, 21.2, 20.2, 20.1, 4.4, 4.4. ; **^{19}F NMR** (471 MHz, $CDCl_3$): δ (ppm) = -145.78 (m). **^{11}B-NMR** (160 MHz, $CDCl_3$): δ (ppm) = 0.32 (t, J_{B-F} = 28.9 Hz). **HRLSI-MS** (MALDI-TOF): calculated for $C_{78}H_{63}B_3F_6N_6$: 1230.5295, Found: 1230.5304, ppm error = 2.8.; **UV/vis ($CHCl_3$)**: λ_{max} (nm) (log ε) = 601 (3.5). **Φ_F** (exc. λ = 510 nm) = 0.28.; **FT-IR**: ν (cm^{-1}) = 3063, 2897, 1550, 1413, 1135, 1107, 1092, 798. **Mp** > 200 °C.

BODIPY **4**

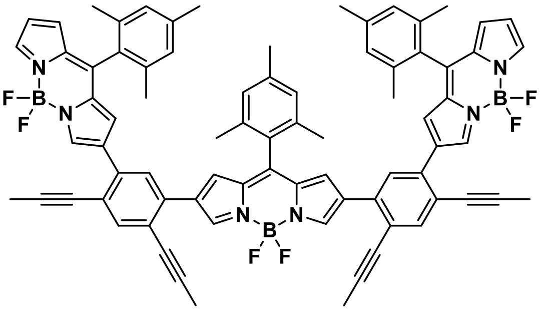

General procedure A was employed with the following quantities: **I2** (75 mg, 13.9 mmol), **B** (35.5 mg, 63 μmol), ^{t}BuXPhos Pd G3 (10 mg, 12.4 μmol) and K_3PO_4 (59 mg, 37.7 mmol). The crude product was purified by column chromatography (0–5 % ethyl acetate in toluene) to afford **4** (25mg, 32%) as a blue solid. **^{1}H-NMR** (500 MHz, $CDCl_3$): δ (ppm) = 8.49 (s, 2H), 8.48 (s, 2H), 7.98 (s, 2H), 7.56 (s, 2H), 7.44 (s, 2H), 7.15 (s, 2H), 7.14 (s, 2H), 7.04 (s, 2H), 7.03 (s, 4H), 6.75 (d, *J* = 4.0 Hz, 2H), 6.55-6.54 (m, 2H), 2.42 (s, 9H), 2.25 (s, 6H), 2.20 (s, 12H), 2.01 (s, 6H), 2.00 (s, 6H).; **^{13}C-NMR** (126 MHz, $CDCl_3$): δ (ppm) = 144.4, 144.1, 138.3, 138.1, 136.4, 133.7, 133.7, 132.8, 130.1, 129.7, 128.4, 128.3, 127.3, 126.0, 91.6, 79.0, 21.2, 21.2, 20.2, 20.1, 4.3, 4.3. ; **^{19}F NMR** (471 MHz, $CDCl_3$): δ (ppm) = -145.68 (m). **^{11}B-NMR** (160 MHz, $CDCl_3$): δ (ppm) = 0.3 (t, *J* = 29.1 Hz). **HRLSI-MS** (MALDI-TOF): calculated for $C_{78}H_{63}B_3F_6N_6$: 1230.5295, Found: 1230.5311, ppm error = 3.1; **UV/vis ($CHCl_3$)**: λ_{max} (nm) (log ε) = 550 (3.9), 583 (3.7). **Φ_F** (exc. λ = 510 nm) = 0.26. **FT-IR**: ν (cm^{-1}) = 3044, 2912, 1551, 1433, 1129, 1107, 1067, 788.; **Mp** > 200 °C.

Synthesis and characterization of BODIPY-anthracene oligomers

BODIPY **DIM-para**

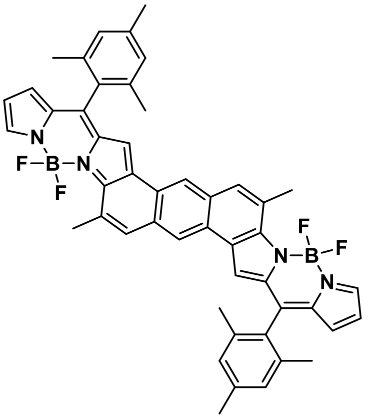

In a Schlenk tube **1** (0.013 mmol,1 eq), $PPh^{F}_{3}AuCl$ (0.0013 mmol, 0.1 eq), $AgSbF_6$ (0.0013 mmol, 0.1 eq) were dissolved in DCM (1 mL) under argon atmosphere. The reaction was stirred at 40 °C for 1h. After that, the crude of the reaction was purified by column chromatography on silica gel using DCM as eluent. The product obtained from the column was subjected to size exclusion chromatography using $CHCl_3$ as eluent. The product was recrystallized from a DCM/hexane mixture to afford **DIM-para** as a green solid in 93% yield. **^{1}H-NMR** (500 MHz, $CDCl_3$): δ (ppm) = 8.25 (s, 2H), 8.04 (s, 2H), 7.60 (s, 2H), 7.40 (s, 2H), 7.06 (s, 4H), 6.74 (d, *J* = 4.0 Hz, 2H), 6.59 (d, *J* = 4.1 Hz, 2H), 2.92 (s, 6H), 2.45 (s, 6H), 2.17 (s, 12H).; **^{13}C-NMR** (126 MHz, $CDCl_3$): δ (ppm) = 146.59, 145.64, 139.04, 137.99, 136.80, 135.67, 133.78, 130.31, 129.68, 129.38, 128.31, 127.90, 125.53, 122.62, 121.47, 120.20, 21.27, 20.70, 20.18.; **^{19}F-NMR** (282 MHz, $CDCl_3$): δ (ppm) = - 134.72 (m); **^{11}B-NMR** (128 MHz, $CDCl_3$): δ (ppm)

= 1.12 (t, J_{B-F} = 35 Hz); **HRLSI-MS** (MALDI-TOF): calculated for: $C_{48}H_{40}B_2F_4N_4$: 770.3375, Found: 770.3379, ppm error = 1.4.; **UV/vis (Toluene)**: λ_{max} (nm) (log ε) = 694 (5.4). **FT-IR**: ν (cm^{-1}) = 3069, 3024, 3010, 2910, 1544, 1430, 1223, 1097, 811, 642. **Mp** > 200 ºC.

BODIPY **DIM-meta**

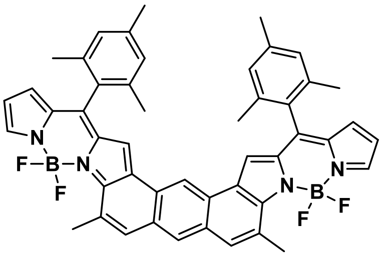

In a Schlenk tube **2** (0.013 mmol,1 eq), $PPh^{F}_{3}AuCl$ (0.0013 mmol, 0.1 eq), $AgSbF_6$ (0.0013 mmol, 0.1 eq) were dissolved in DCM (1 mL) under argon atmosphere. The reaction was stirred at 40 ºC for 1h. After that, the crude of the reaction was purified by column chromatography on silica gel using DCM as eluent. The product obtained from the column was subjected to size exclusion chromatography using $CHCl_3$ as eluent. The product was recrystallized from a DCM/hexane mixture to afford **DIM-meta** as a blue solid in 91% yield. **^{1}H-NMR** (500 MHz, $CDCl_3$): δ (ppm) = 8.43 (s, 1H), 8.04 (s, 2H), 8.00 (s, 2H), 7.56 (s, 2H), 7.43 (s, 2H), 7.06 (s, 4H), 6.71 (d, *J* = 4.0 Hz, 2H), 6.58 (d, *J* = 4.1 Hz, 2H), 2.94 (s, 6H), 2.44 (s, 6H), 2.18 (s, 12H); **^{13}C-NMR** (126 MHz, $CDCl_3$): δ (ppm) = 151.36, 146.53, 145.59, 139.07, 137.69, 136.80, 136.71, 136.29, 130.45, 129.84, 129.58, 128.78, 128.48, 128.11, 127.16, 126.36, 121.77, 120.19, 117.70, 21.26, 20.60, 20.21.; **^{19}F-NMR** (282 MHz, $CDCl_3$): δ (ppm) = - 133.51 (m); **^{11}B-NMR** (128 MHz, $CDCl_3$): δ (ppm) = 1.06 (t, J_{B-F} = 35 Hz); **HRLSI-MS** (MALDI-TOF): calculated for: $C_{48}H_{40}B_2F_4N_4$: 770.3375, Found: 770.3382, ppm error = 1.3.; **UV/vis (Toluene)**: λ_{max} (nm) (log ε) = 691 (5.3). ; **FT-IR**: ν (cm^{-1}) = 3071, 3018, 3010, 2903, 1546, 1430, 1222, 1092, 801, 630. **Mp** > 200 ºC.

BODIPY **TRIM-para**

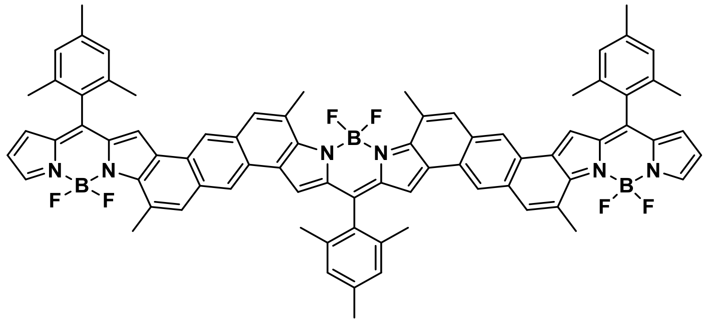

In a Schlenk tube **3** (0.013 mmol,1 eq), $PPh^{F}_{3}AuCl$ (0.0013 mmol, 0.1 eq), $AgSbF_6$ (0.0013 mmol, 0.1 eq) were dissolved in DCM (1 mL) under argon atmosphere. The reaction was stirred at 40 ºC for 4h. After that, the crude of the reaction was purified by column chromatography on silica gel using DCM as eluent. The product obtained from the column was subjected to size exclusion chromatography using $CHCl_3$ as eluent. The product was recrystallized from a DCM/hexane mixture to afford **TRIM-para** as a black solid in 67% yield. **^{1}H-NMR** (500 MHz, $CDCl_3$): δ (ppm) = 8.28 (s, 2H), 8.24 (s, 2H), 8.05 (s, 2H), 7.61 (s, 4H), 7.41 (d, 4H), 7.15 (s, 2H), 7.06 (s, 4H), 6.76 (d, *J* = 4.1 Hz, 2H), 6.58 (d, *J* = 4.1 Hz, 2H), 2.99 (s, 6H), 2.93 (s, 6H), 2.54 (s, 3H), 2.46 (s, 6H), 2.24 (s, 6H), 2.18 (s, 12H).; **^{13}C-NMR** (126 MHz, $CDCl_3$): δ (ppm) = 151.70, 150.77, 146.78, 145.81, 139.06, 137.86, 137.34, 136.80, 136.72, 135.65, 131.23, 130.31, 129.77, 129.47, 128.32, 128.03, 125.75, 125.46, 123.02, 122.84, 121.44,

120.34, 22.71, 21.37, 21.27, 20.72, 20.38, 20.19.; **^{19}F NMR** (471 MHz, $CDCl_3$): δ (ppm) = -123.68 (m), -134.67 (m); **^{11}B-NMR** (160 MHz, $CDCl_3$): δ (ppm) = -1.36 (m); **HRLSI-MS** (MALDI-TOF): calculated for $C_{78}H_{63}B_3F_6N_6$: 1230.5295, Found: 1230.5313, ppm error = 1.5.; **UV/vis (Toluene)**: λ_{max} (nm) (log ε) = 807 (5.7). **FT-IR**: ν (cm^{-1}) = 3032, 2926, 1433, 1131, 1117, 1059, 787.; **Mp** > 200 ºC.

BODIPY **TRIM-meta**

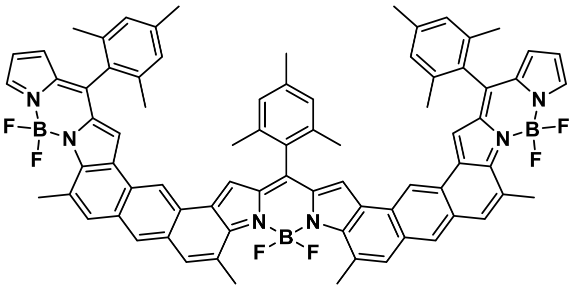

In a Schlenk tube **4** (0.013 mmol,1 eq), $PPh^F{}_3AuCl$ (0.0013 mmol, 0.1 eq), $AgSbF_6$ (0.0013 mmol, 0.1 eq) were dissolved in DCM (1 mL) under argon atmosphere. The reaction was stirred at 40 ºC for 4h. After that, the crude of the reaction was purified by column chromatography on silica gel using DCM as eluent. The product obtained from the column was subjected to size exclusion chromatography using $CHCl_3$ as eluent. The product was recrystallized from a DCM/hexane mixture to afford **TRIM-para** as a dark blue solid in 72% yield. **^{1}H-NMR** (500 MHz, $CDCl_3$): δ (ppm) = 8.47 (s, 2H), 8.08 (s, 2H), 8.02 (s, 2H), 7.71 (d, 4H), 7.46 (s, 2H), 7.42 (s, 2H), 7.19 (s, 2H), 7.11 (s, 4H) 6.75 (d, *J* = 4.1 Hz, 2H), 6.62 (d, *J* = 4.1 Hz, 2H), 3.03 (s, 6H), 2.97 (s, 6H), 2.54 (s, 3H), 2.49 (s, 6H), 2.28 (s, 6H), 2.22 (s, 12H).; **^{13}C-NMR** (126 MHz, $CDCl_3$): δ (ppm) = 151.31, 146.53, 145.77, 139.04, 137.30, 136.81, 136.24, 130.45, 128.95, 128.84, 128.67, 128.47, 128.30, 127.59, 126.24, 121.70, 120.50, 120.27, 118.05, 21.32, 21.22, 20.58, 20.41, 20.21.; **^{19}F NMR** (471 MHz, $CDCl_3$): δ (ppm) = -122.57 (m), -133.55 (m); **^{11}B-NMR** (160 MHz, $CDCl_3$): δ (ppm) = -1.04 (t, J_{B-F} = 35 Hz), -1.73 (t, J_{B-F} = 35 Hz); **HRLSI-MS** (MALDI-TOF): calculated for $C_{78}H_{63}B_3F_6N_6$: 1230.5295, Found: 1230.5321, ppm error = 2.1.; **UV/vis (Toluene)**: λ_{max} (nm) (log ε) = 802 (5.7). **FT-IR**: ν (cm^{-1}) = 3032, 2926, 1433, 1131, 1117, 1059, 787; **Mp** > 200 ºC.

X-Ray Crystal Structures and Crystallographic Details

Crystal packing of BODIPYs **DIM-para and DIM-meta**.

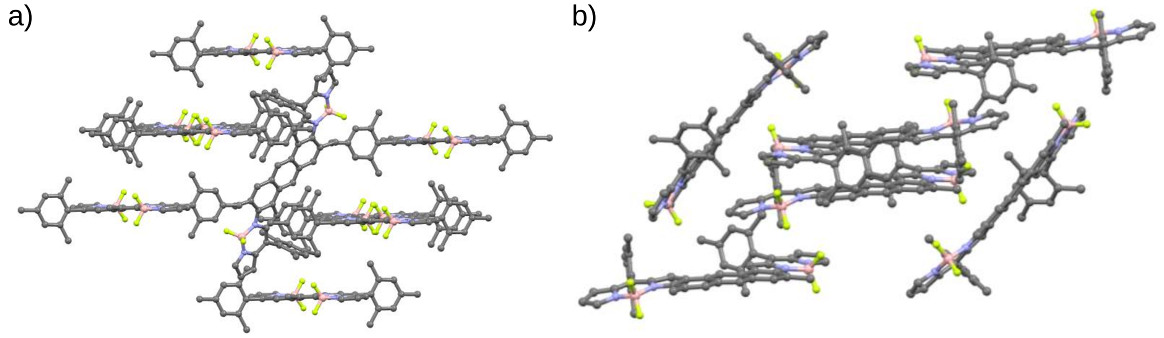

Figure S3.1. Crystal packing of a) **DIM-para** and b) **DIM-meta.** Hydrogen atoms and solvent molecules are omitted for clarity.

Table S3.1. Selected crystallographic data for **DIM-para**. CCDC number= 2130682

**Chemical formula**	$C_{50}H_{42}B_2Cl_6F_4N_4$	
**Formula weight**	1009.19 g/mol	
**Temperature**	150(2) K	
**Wavelength**	0.71073 Å	
**Crystal size**	0.080 x 0.217 x 0.375 mm	
**Crystal habit**	dark brown-red prismatic	
**Crystal system**	monoclinic	
**Space group**	P 1 21/n 1	
**Unit cell dimensions**	a = 12.3932(3) Å	α = 90°
	b = 11.8450(3) Å	β = 109.7595(10)°
	c = 17.4465(4) Å	γ = 90°
**Volume**	2410.30(10) $Å^3$	
**Z**	2	
**Density (calculated)**	1.391 g/cm^3	
**Absorption coefficient**	0.412 mm^{-1}	
**F(000)**	1036	
**Theta range for data collection**	2.12 to 25.34°	
**Index ranges**	-14<=h<=14, -14<=k<=14, -21<=l<=20	
**Reflections collected**	30577	
**Independent reflections**	4399 [R(int) = 0.0249]	
**Coverage of independent reflections**	99.8%	
**Absorption correction**	multi-scan	
**Max. and min. transmission**	0.9680 and 0.8610	
**Structure solution technique**	direct methods	
**Structure solution program**	SHELXS-97 (Sheldrick 2008)	
**Refinement method**	Full-matrix least-squares on F2	
**Refinement program**	SHELXL-2014/7 (Sheldrick, 2014)	
**Function minimized**	Σ w(Fo2 - Fc2)2	
**Data / restraints / parameters**	4399 / 12 / 300	
**Goodness-of-fit on F2**	1.050	
**Final R indices**	3789 data; I>2σ(I)	R1 = 0.1216, wR2 = 0.3242
	all data	R1 = 0.1347, wR2 = 0.3393
**Weighting scheme**	$w=1/[\sigma^2(F_o^2)+(0.1804P)^2+15.5389P]$ where $P=(F_o^2+2F_c^2)/3$	
**Largest diff. peak and hole**	2.125 and -1.879 $eÅ^{-3}$	
**R.M.S. deviation from mean**	0.142 $eÅ^{-3}$	

Table S3.2. Selected crystallographic data for **DIM-meta**. CCDC number= 2130683

**Chemical formula**	$C_{51}H_{47}B_2F_4N_4$	
**Formula weight**	813.54 g/mol	
**Temperature**	250(2) K	
**Wavelength**	0.71073 Å	
**Crystal size**	0.047 x 0.184 x 0.265 mm	
**Crystal habit**	dark purple plate	
**Crystal system**	monoclinic	
**Space group**	P 1 21/c 1	
**Unit cell dimensions**	a = 16.5075(9) Å	α = 90°
	b = 14.3818(8) Å	β = 96.608(2)°
	c = 19.5801(11) Å	γ = 90°
**Volume**	4617.6(4) $Å^3$	
**Z**	4	
**Density (calculated)**	1.170 g/cm^3	
**Absorption coefficient**	0.079 mm^{-1}	

**F(000)**	1708	
**Theta range for data collection**	1.24 to 25.35°	
**Index ranges**	-19<=h<=19, -17<=k<=17, -23<=l<=21	
**Reflections collected**	83378	
**Independent reflections**	8444 [R(int) = 0.0708]	
**Coverage of independent reflections**	99.9%	
**Absorption correction**	multi-scan	
**Max. and min. transmission**	0.9960 and 0.9790	
**Refinement method**	Full-matrix least-squares on F2	
**Refinement program**	SHELXL-2014/7 (Sheldrick, 2014)	
**Function minimized**	Σ w(Fo2 - Fc2)2	
**Data / restraints / parameters**	8444 / 5 / 557	
**Goodness-of-fit on F2**	1.064	
**Final R indices**	4829 data; I>2σ(I)	R1 = 0.0754, wR2 = 0.2172
	all data	R1 = 0.1360, wR2 = 0.2709
**Weighting scheme**	$w=1/[\sigma^2(F_o^2)+(0.1573P)^2+1.5571P]$ where $P=(F_o^2+2F_c^2)/3$	
**Largest diff. peak and hole**	1.087 and -0.367 eÅ^{-3}	
**R.M.S. deviation from mean**	0.147 eÅ^{-3}	

Table S3.3. Selected crystallographic data for **META-meta**. CCDC number= 2130684

**Chemical formula**	$C_{73.25}H_{52.75}B_3Cl_{4.50}F_6N_6O_{0.50}$	
**Formula weight**	1330.92 g/mol	
**Temperature**	250(2) K	
**Wavelength**	0.71073 Å	
**Crystal size**	0.040 x 0.165 x 0.214 mm	
**Crystal habit**	dark purple plate	
**Crystal system**	triclinic	
**Space group**	P -1	
**Unit cell dimensions**	a = 15.468(14) Å	α = 73.71(3)°
	b = 15.922(12) Å	β = 70.49(4)°
	c = 20.541(14) Å	γ = 71.34(3)°
**Volume**	4432.(6) Å^3	
**Z**	2	
**Density (calculated)**	0.997 g/cm^3	
**Absorption coefficient**	0.198 mm^{-1}	
**F(000)**	1368	
**Theta range for data collection**	1.07 to 25.16°	
**Index ranges**	-18<=h<=18, -18<=k<=18, -24<=l<=24	
**Reflections collected**	91398	
**Independent reflections**	15725 [R(int) = 0.1131]	
**Coverage of independent reflections**	98.9%	
**Absorption correction**	Multi-Scan	
**Max. and min. transmission**	0.9920 and 0.9590	
**Structure solution technique**	direct methods	
**Structure solution program**	XT, VERSION 2018/2	
**Refinement method**	Full-matrix least-squares on F2	
**Refinement program**	SHELXL-2018/3 (Sheldrick, 2018)	
**Function minimized**	Σ w(Fo2 - Fc2)2	
**Data / restraints / parameters**	15725 / 3 / 880	
**Goodness-of-fit on F2**	1.019	
**Final R indices**	5953 data; I>2σ(I)	R1 = 0.1724, wR2 = 0.4599
	all data	R1 = 0.3028, wR2 = 0.5752
**Weighting scheme**	$w=1/[\sigma^2(F_o^2)+(0.4046P)^2]$ where $P=(F_o^2+2F_c^2)/3$	

**Absolute structure parameter**	0.00(5)
**Largest diff. peak and hole**	1.351 and -0.520 eÅ^{-3}
**R.M.S. deviation from mean**	0.213 eÅ^{-3}

3. Absorption and Fluorescence Spectra of 1-4 and I1-2

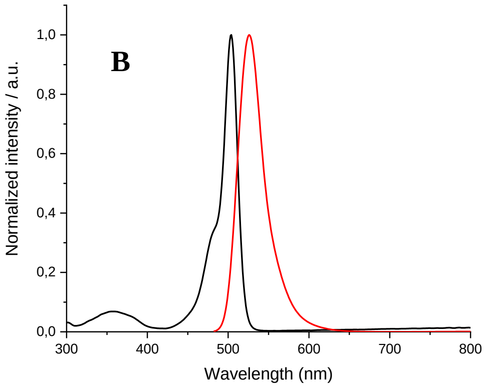

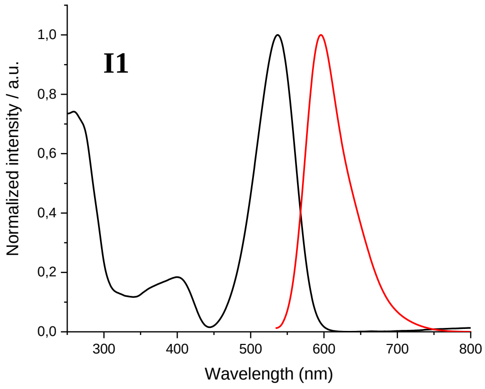

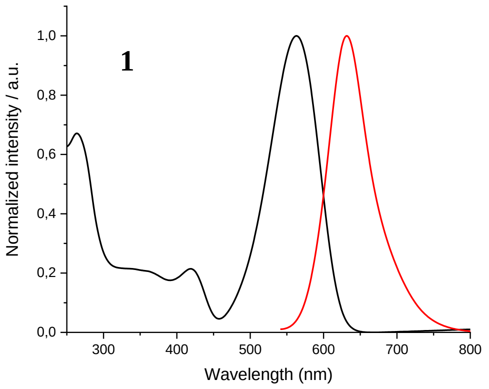

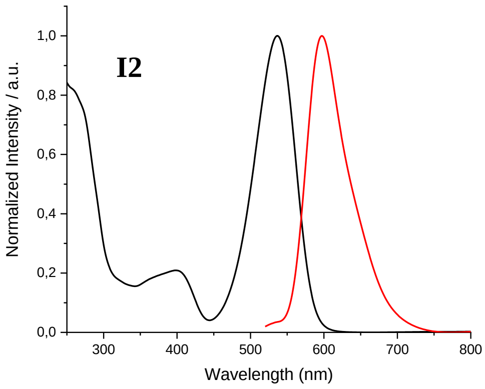

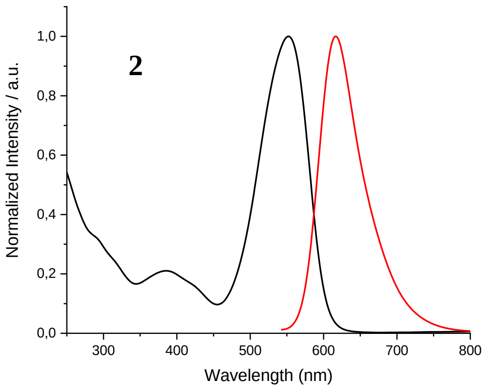

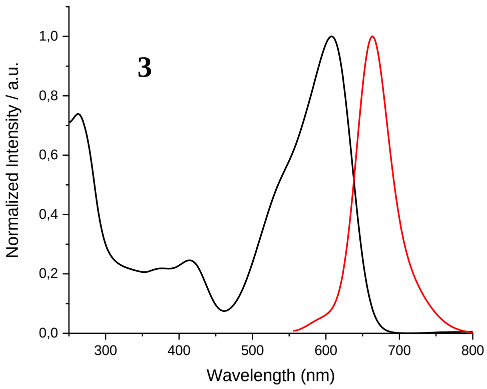

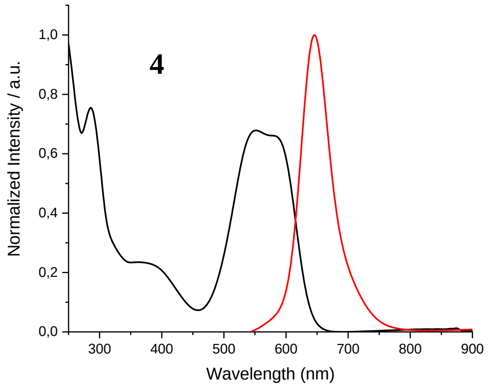

Figure S4.1. Absorption (black) and fluorescence spectra (red) of **B**, **1-4** and **I1-2**.

4. NMR spectra

<u>Boronic ester B</u>

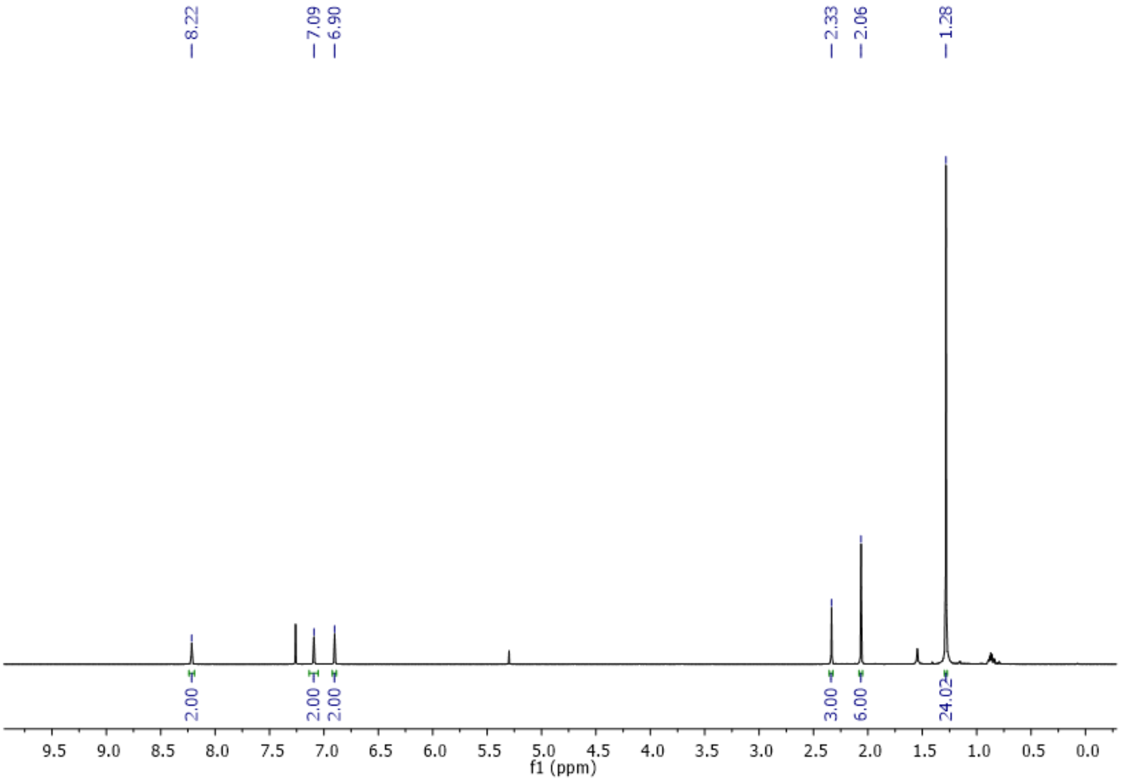

Figure S5.1. ^{1}H-NMR spectrum ($CDCl_3$) of **B**

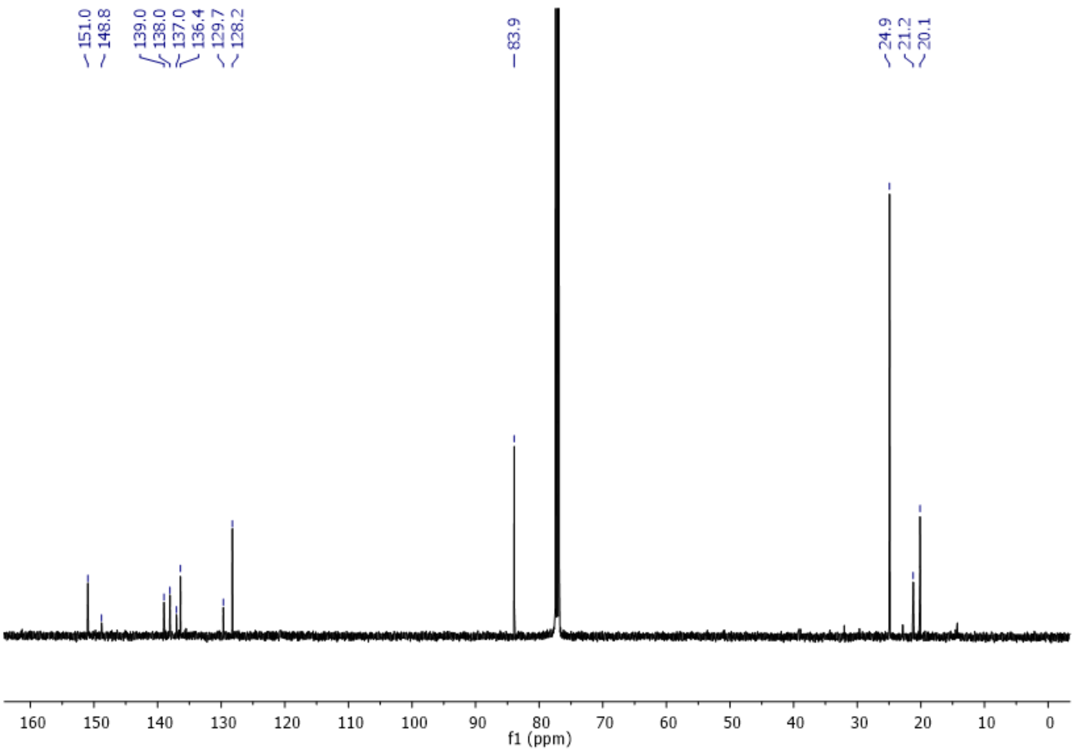

Figure S5.2. ^{13}C-NMR spectrum ($CDCl_3$) of **B**

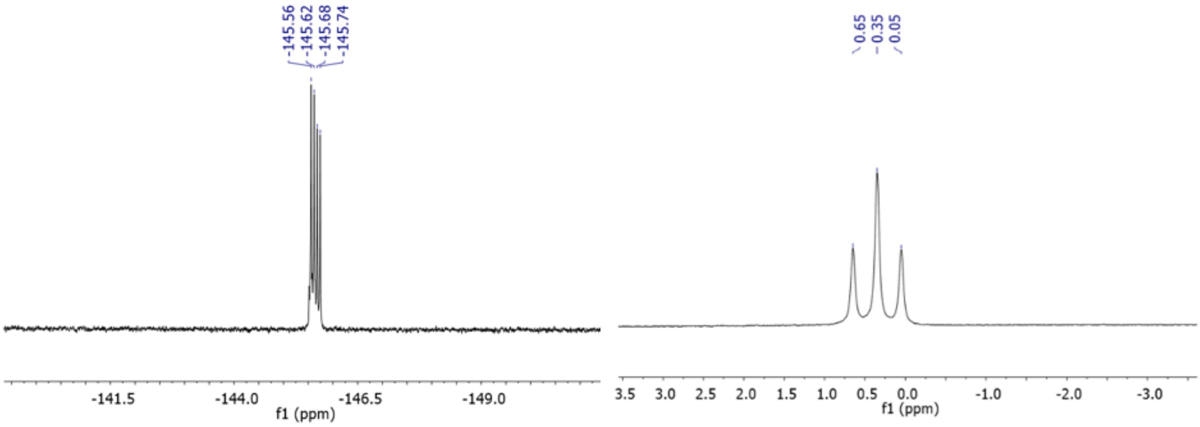

Figure S5.3. ^{19}F-NMR (left) and spectra ^{11}B-NMR (right) ($CDCl_3$) of **B**

BODIPY **I1**

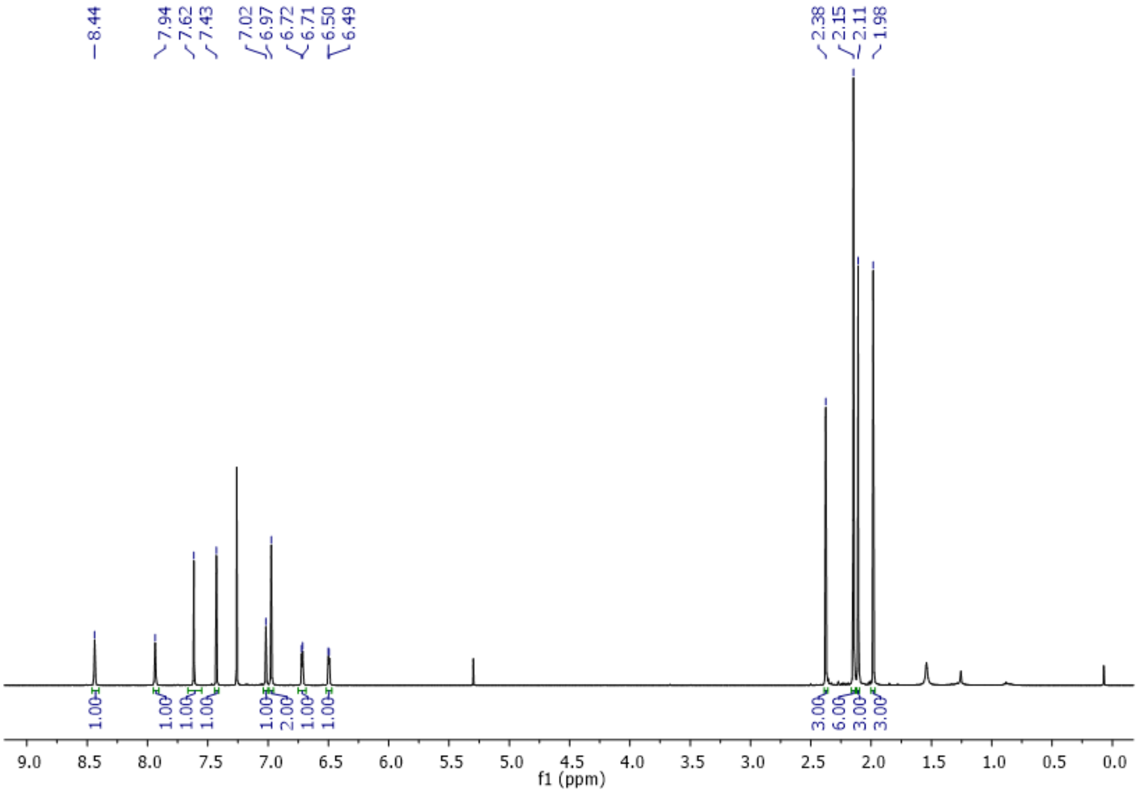

Figure S5.4. ^{1}H-NMR spectrum ($CDCl_3$) of **I1**

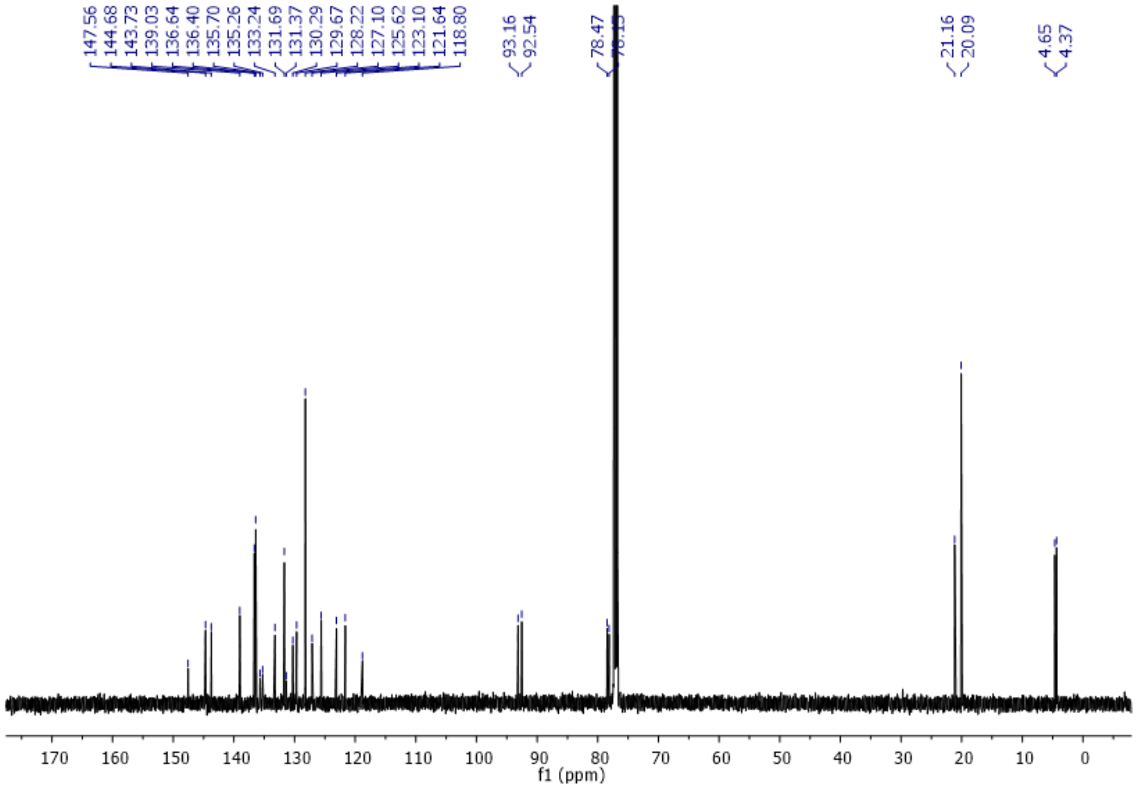

Figure S5.5. ^{13}C-NMR spectrum ($CDCl_3$) of **I1**

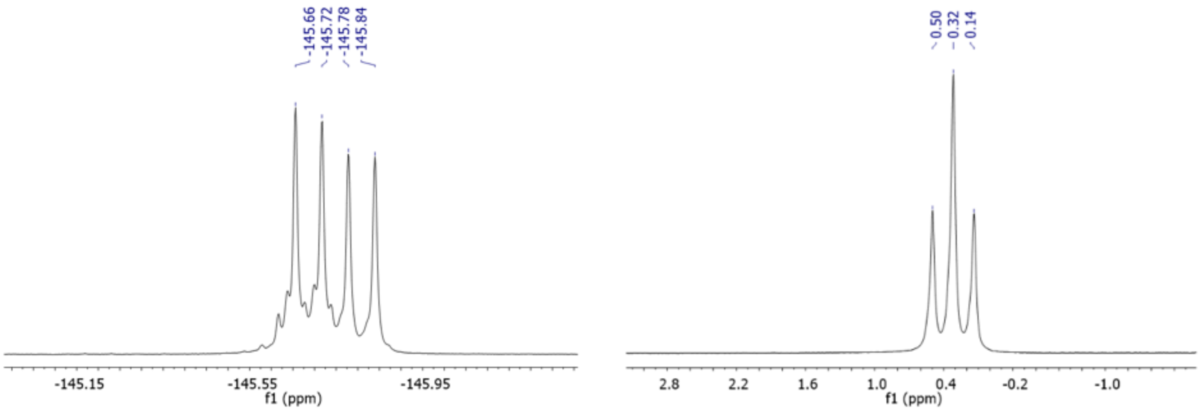

Figure S5.6. ^{19}F-NMR (left) and spectra ^{11}B-NMR (right) ($CDCl_3$) of **I1**

<u>BODIPY **1**</u>

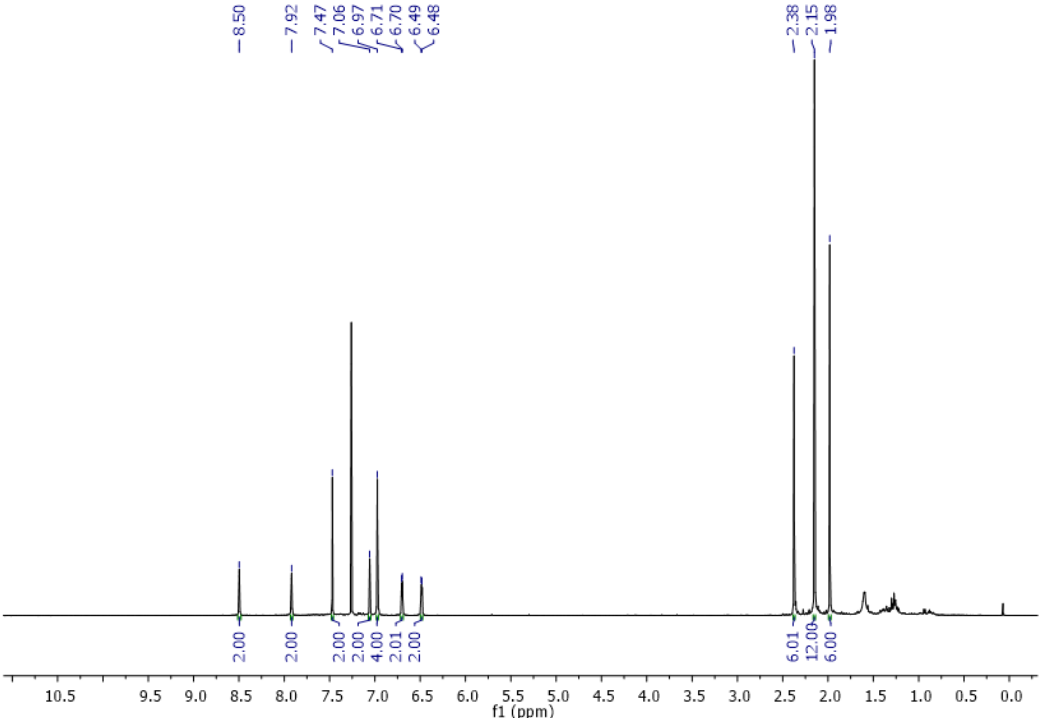

Figure S5.7. ^{1}H-NMR spectrum ($CDCl_3$) of **1**

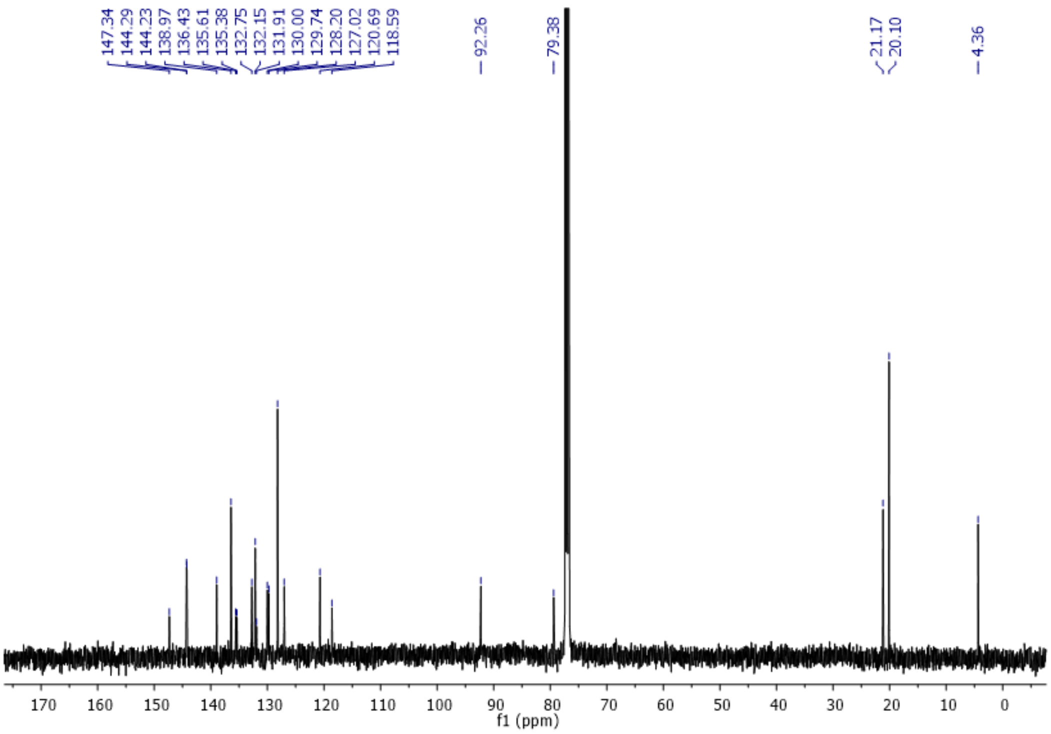

Figure S5.8. ^{13}C-NMR spectrum ($CDCl_3$) of **1**

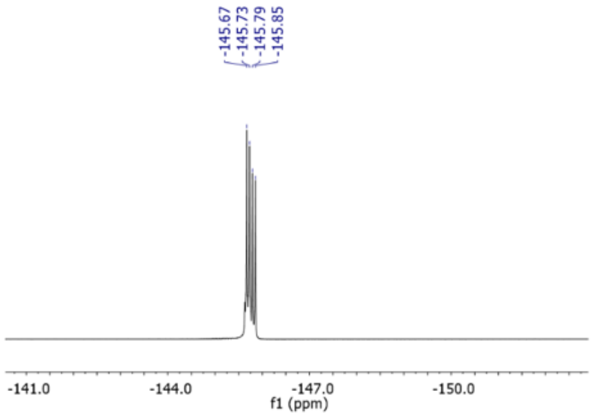

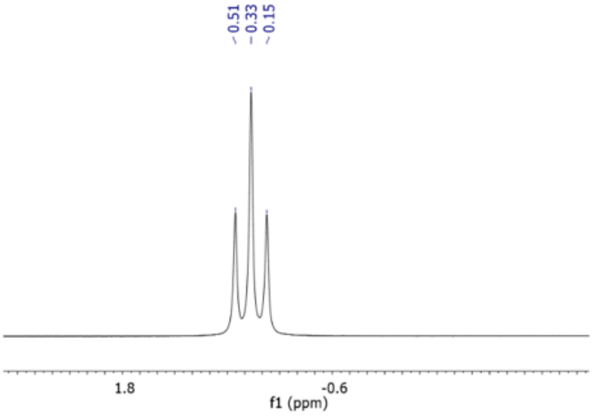

Figure S5.9. ^{19}F-NMR (left) and spectra ^{11}B-NMR (right) ($CDCl_3$) of **1**

BODIPY **I2**

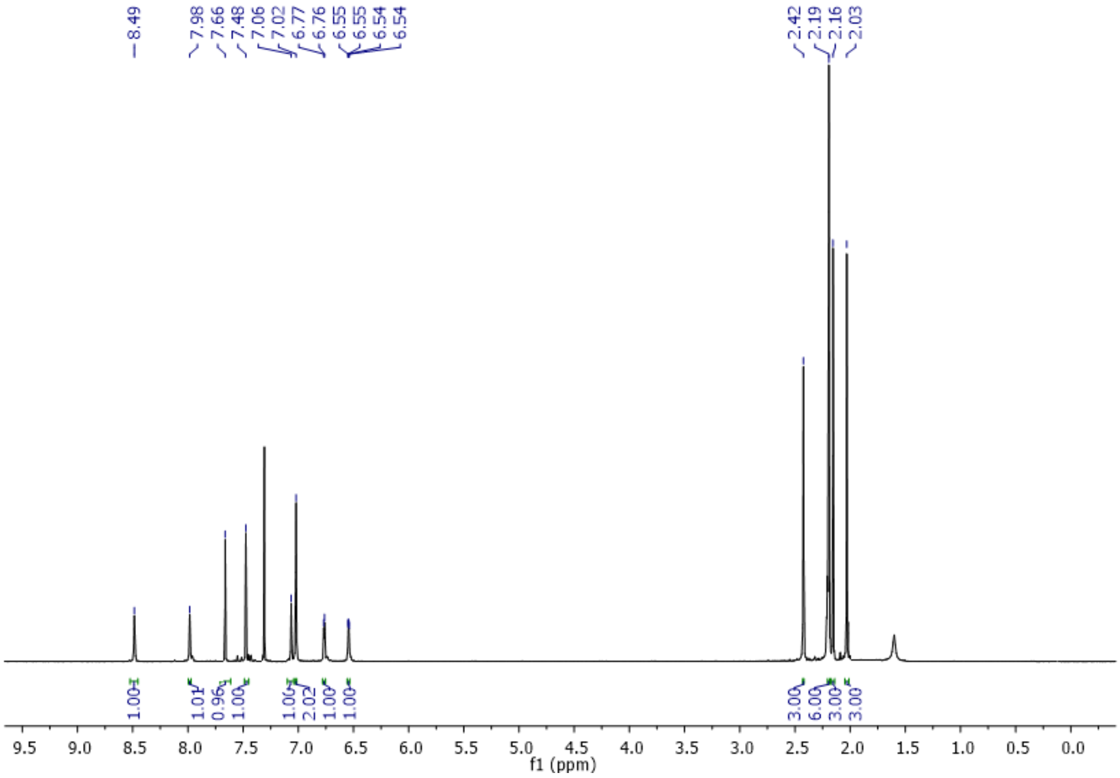

Figure S5.10. ^{1}H-NMR spectrum ($CDCl_3$) of **I2**

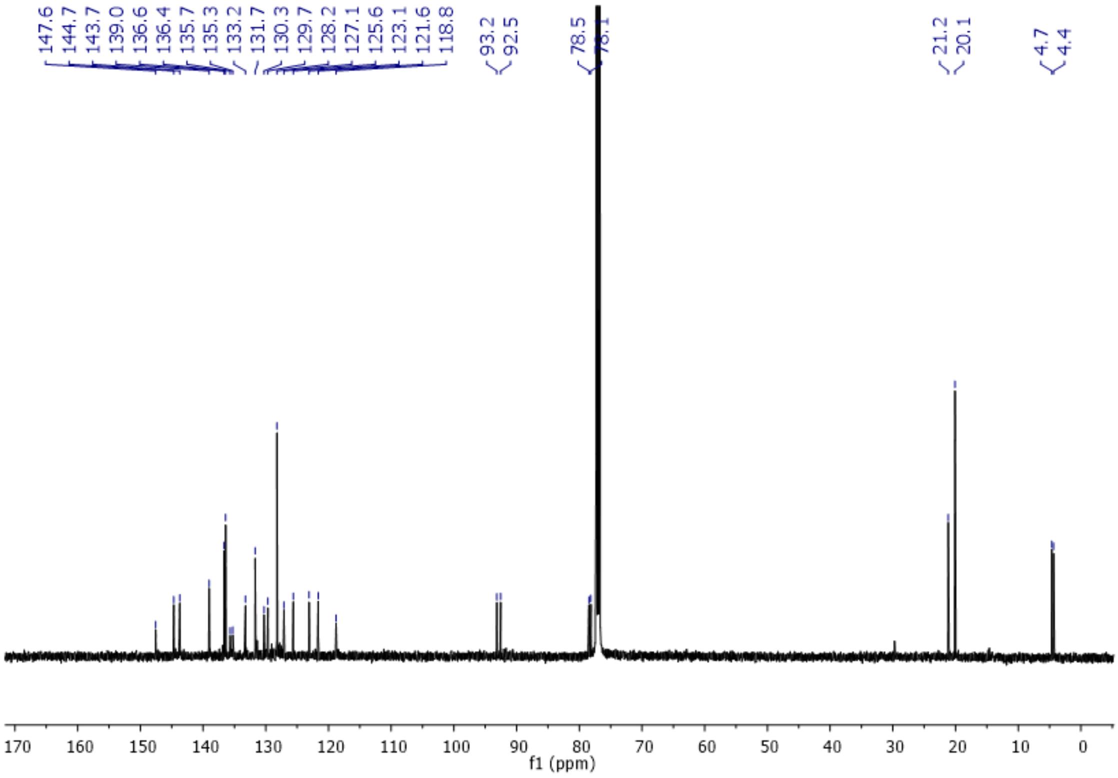

Figure S5.11. ^{13}C-NMR spectrum ($CDCl_3$) of **I2**

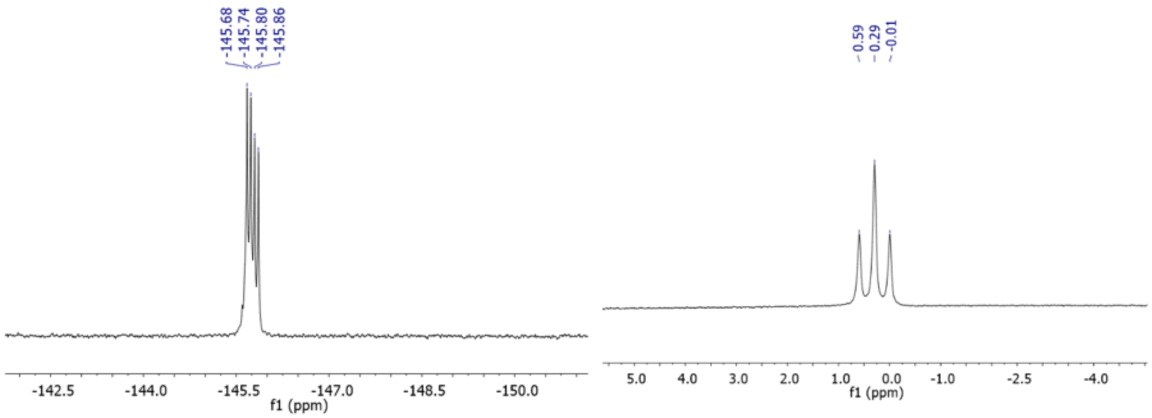

Figure S5.12. ^{19}F-NMR (left) and spectra [11i]B-NMR (right) ($CDCl_3$) of **I2**

<u>BODIPY **2**</u>

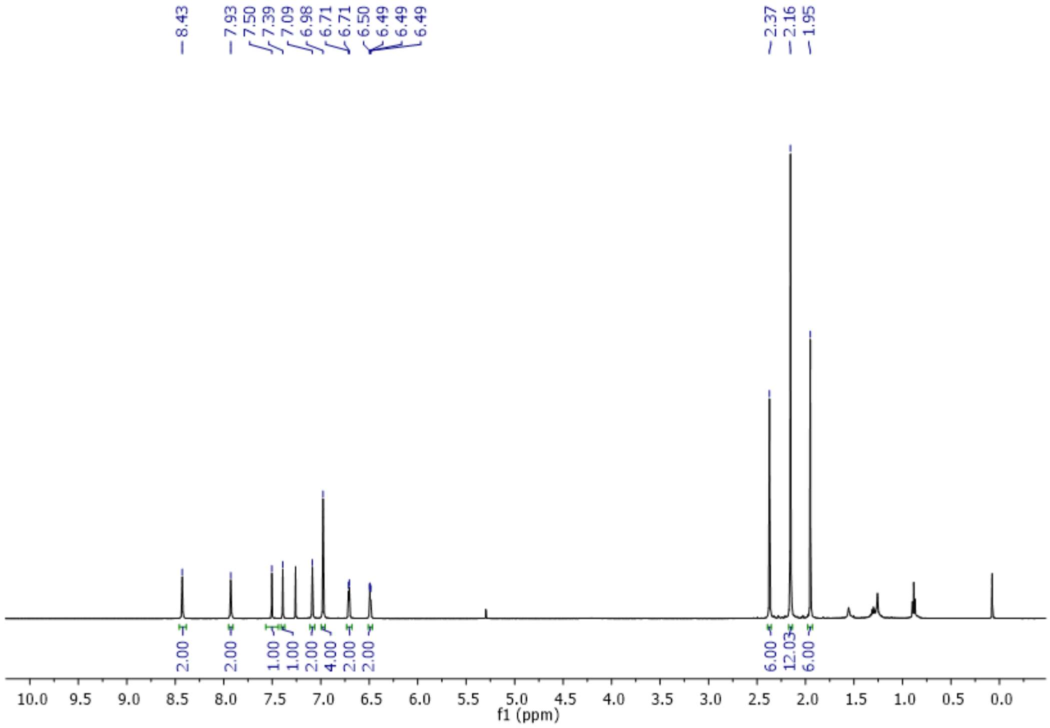

Figure S5.13. ^{1}H-NMR spectrum ($CDCl_3$) of **2**

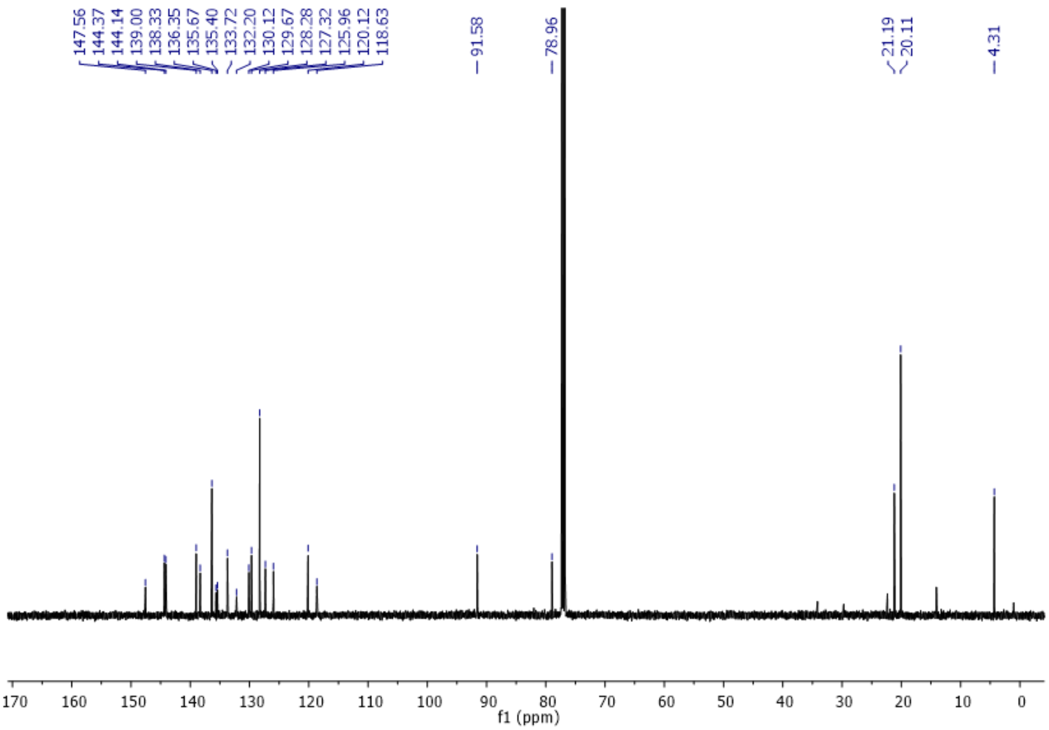

Figure S5.14. ^{13}C-NMR spectrum ($CDCl_3$) of **2**

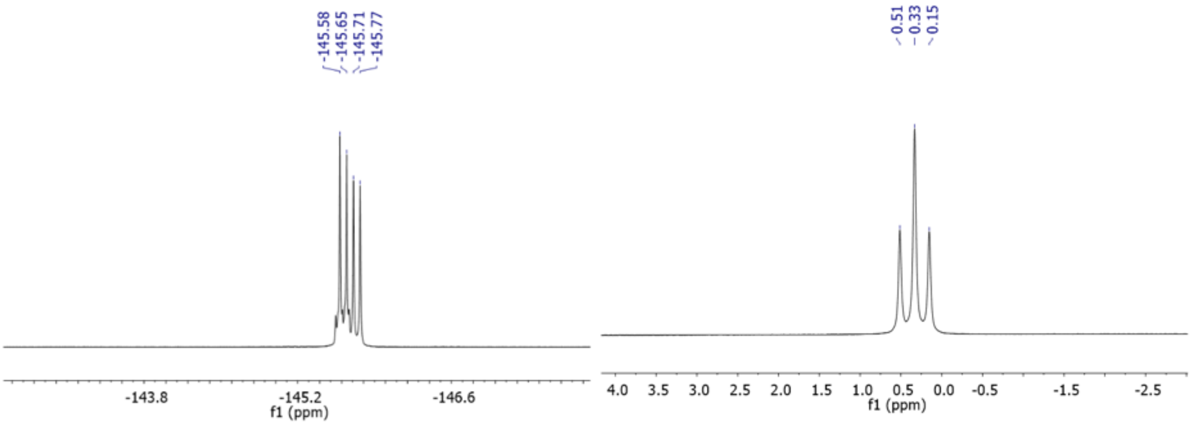

Figure S5.15. ^{19}F-NMR (left) and spectra ^{11}B-NMR (right) ($CDCl_3$) of **2**

BODIPY **3**

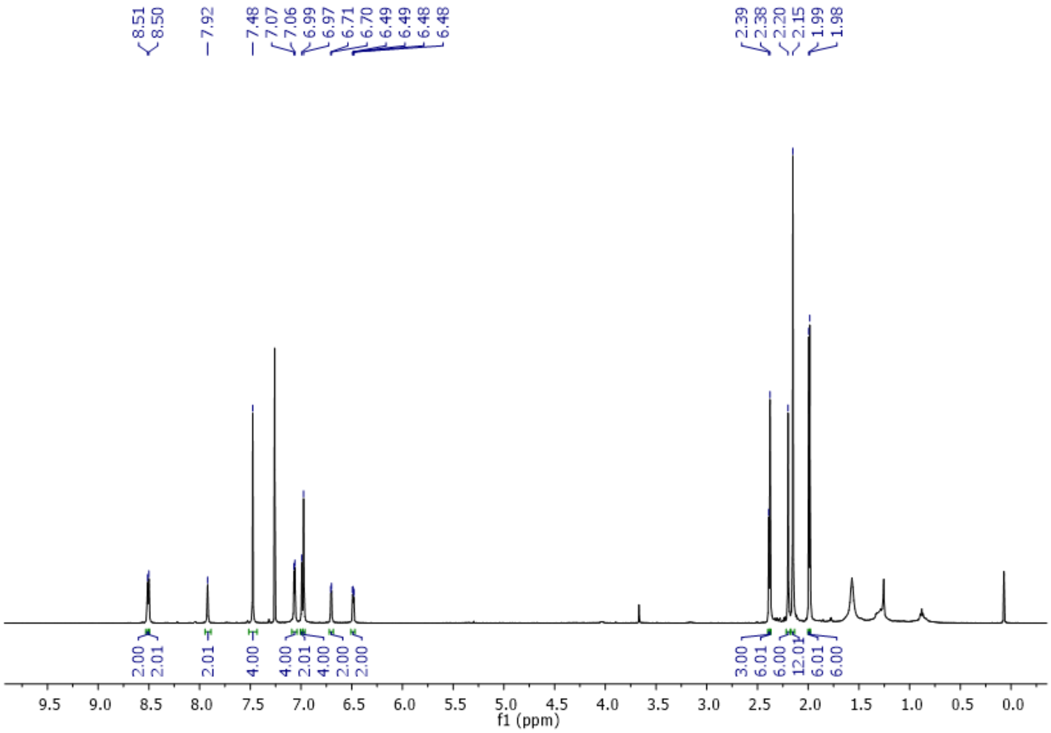

Figure S5.16. ^{1}H-NMR spectrum ($CDCl_3$) of **3**

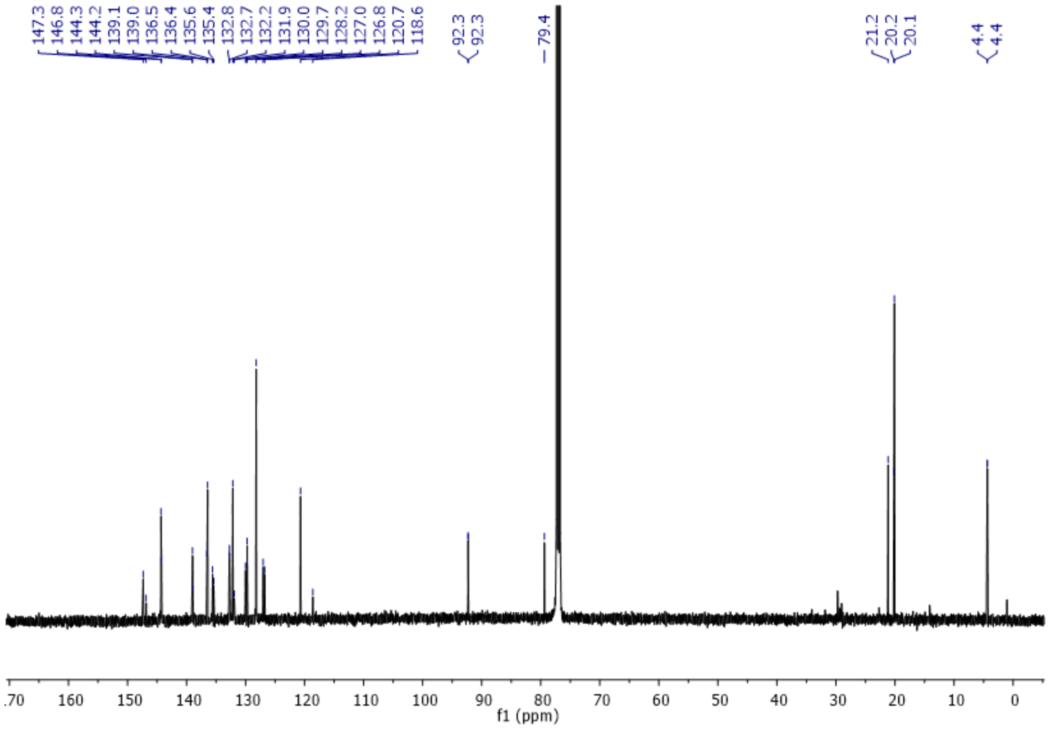

Figure S5.17. ^{13}C-NMR spectrum ($CDCl_3$) of **3**

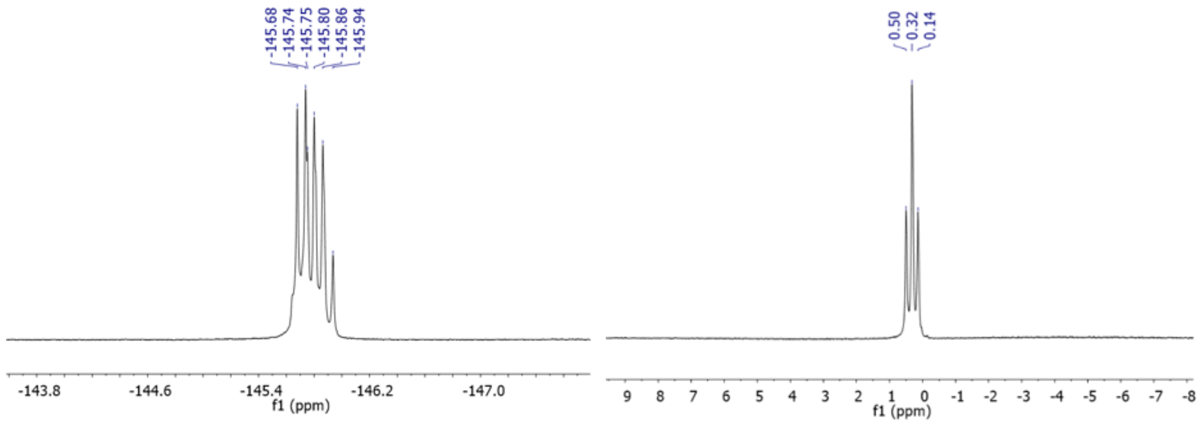

Figure S5.18. ^{19}F-NMR (left) and spectra ^{11}B-NMR (right) ($CDCl_3$) of **3**

<u>BODIPY **4**</u>

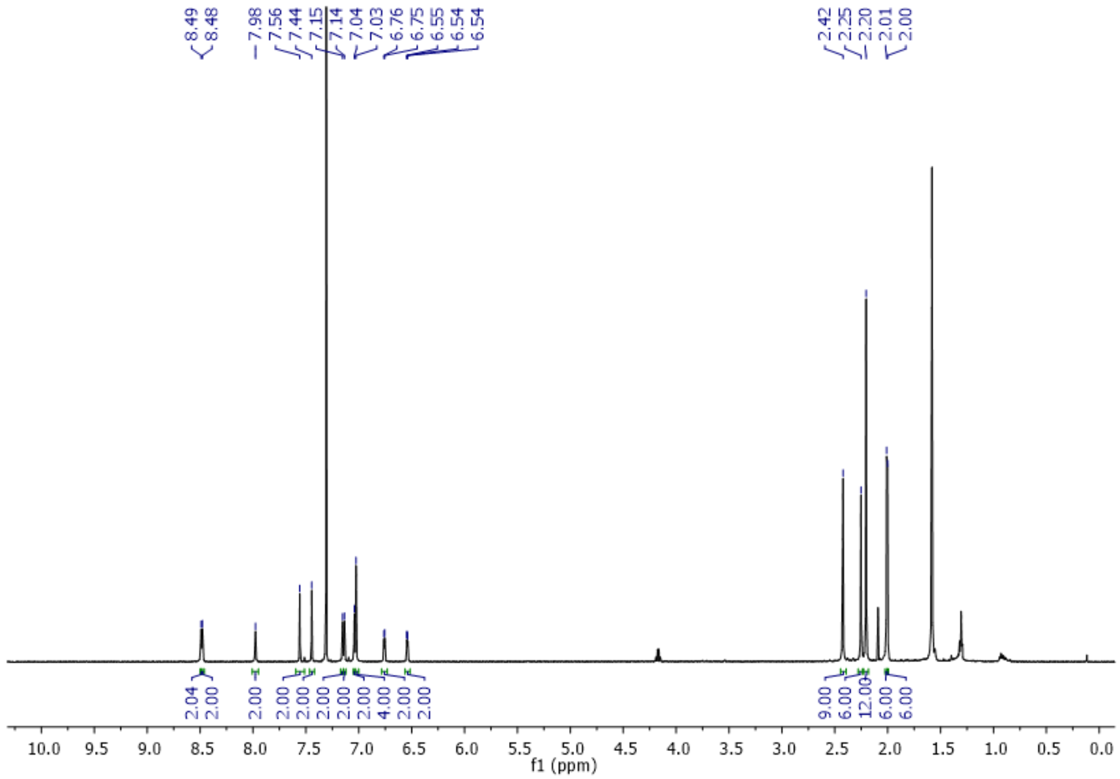

Figure S5.19. ^{1}H-NMR spectrum ($CDCl_3$) of **4**

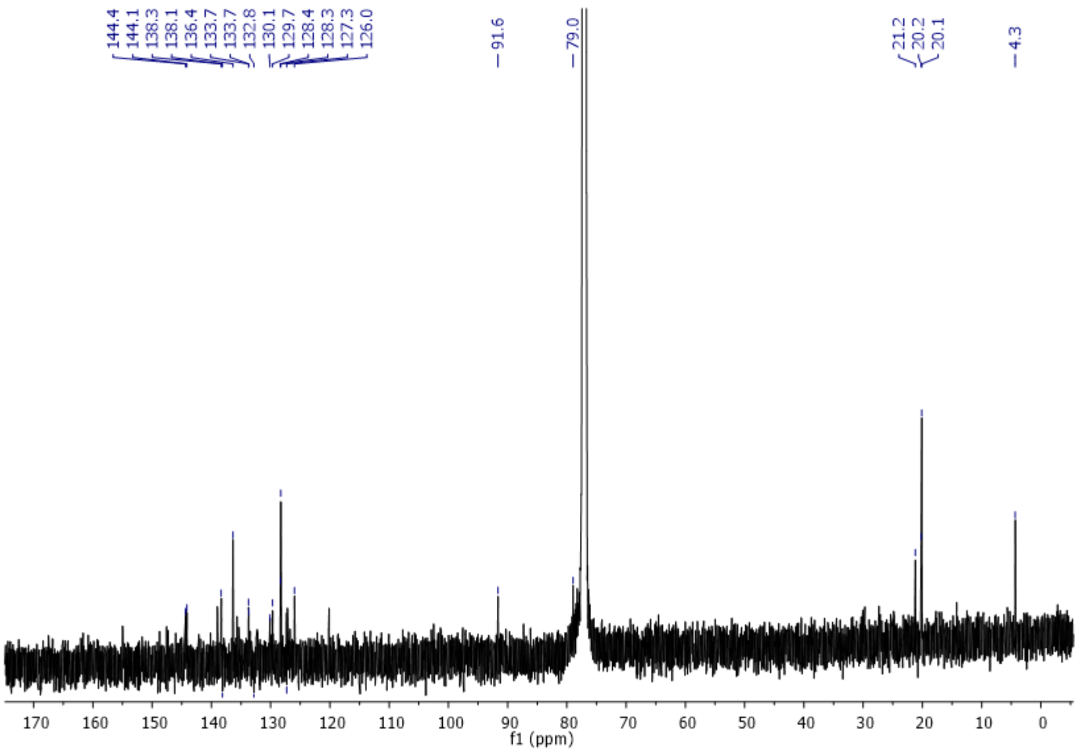

Figure S5.20. ^{13}C-NMR spectrum ($CDCl_3$) of **4**

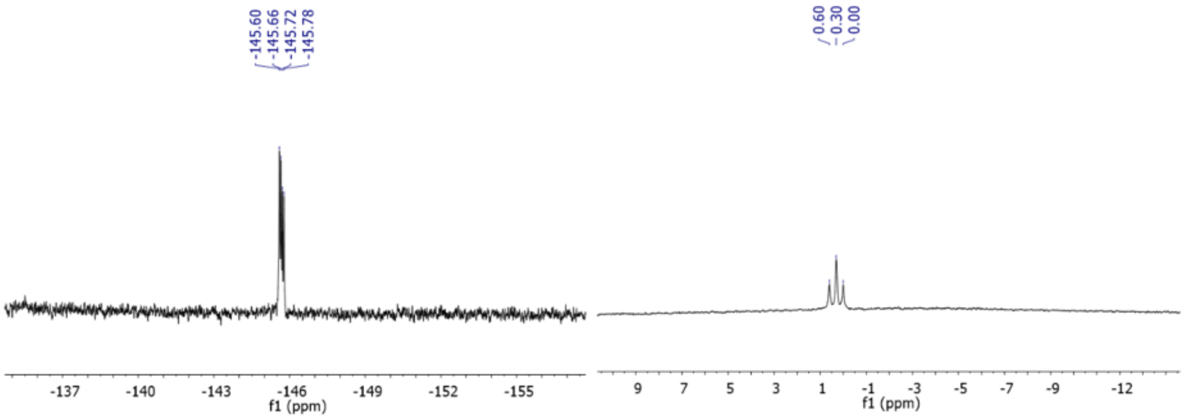

Figure S5.21. ^{19}F-NMR (left) and spectra ^{11}B-NMR (right) ($CDCl_3$) of **4**

BODIPY **DIM-para**

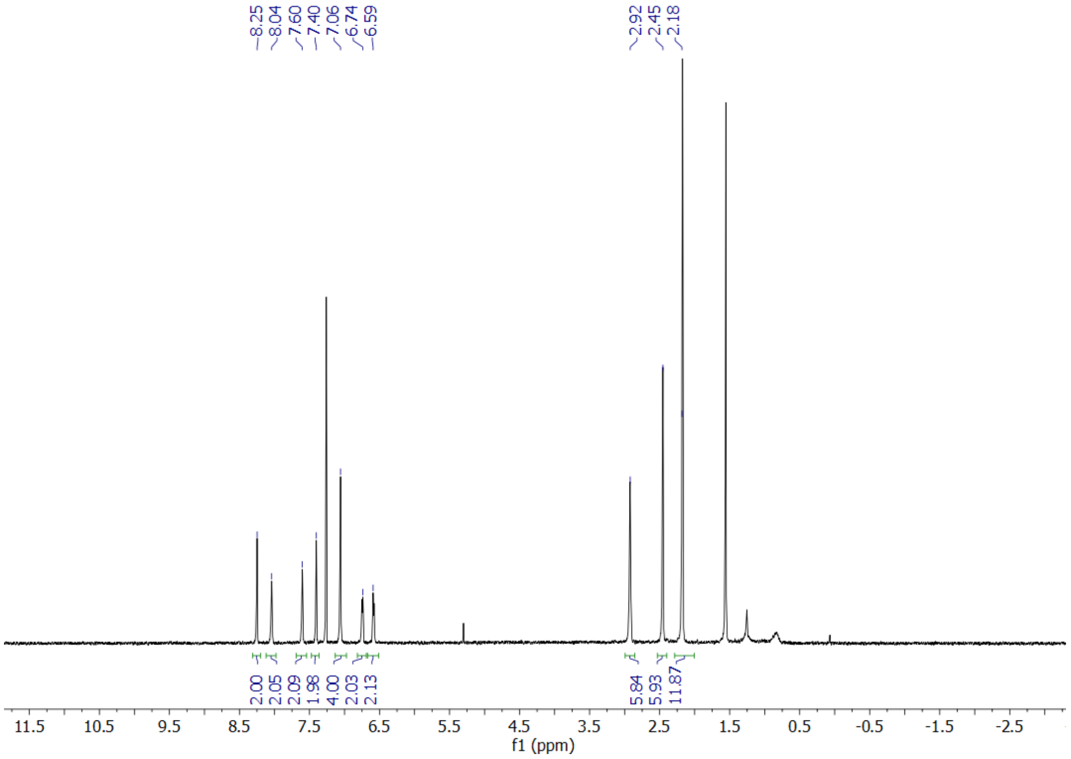

Figure S5.22. ^{1}H-NMR spectrum ($CDCl_3$) of **DIM-para**

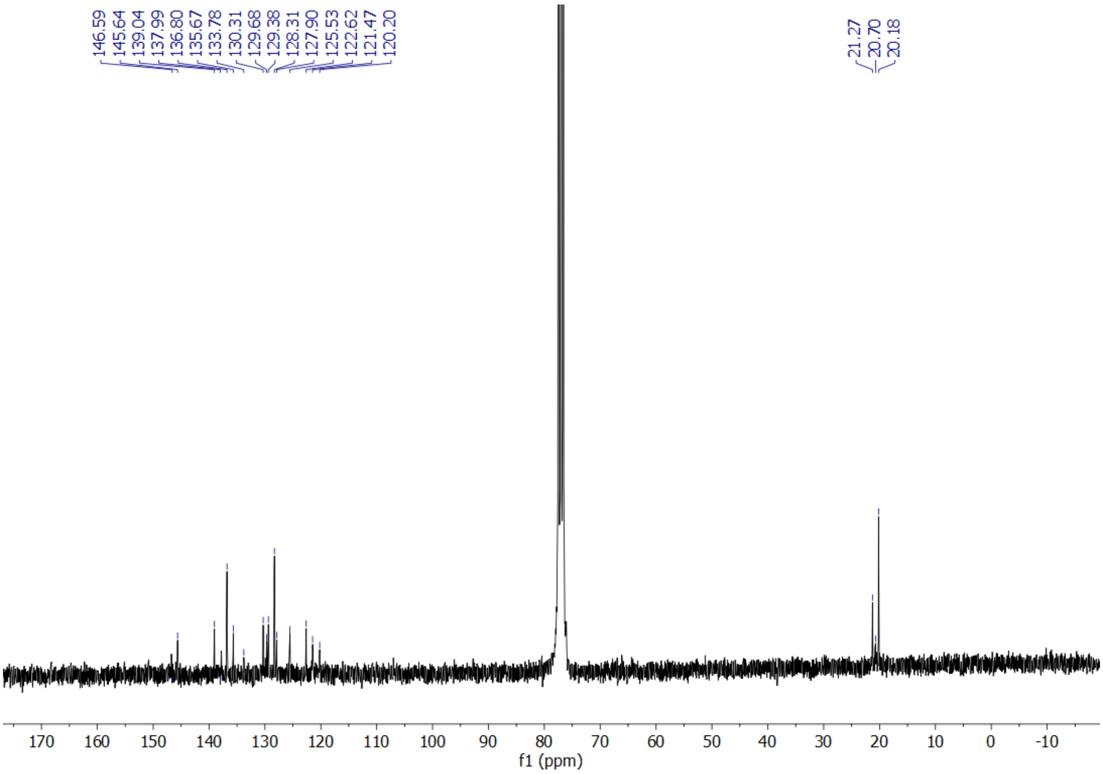

Figure S5.23. ^{13}C-NMR spectrum ($CDCl_3$) of **DIM-para**

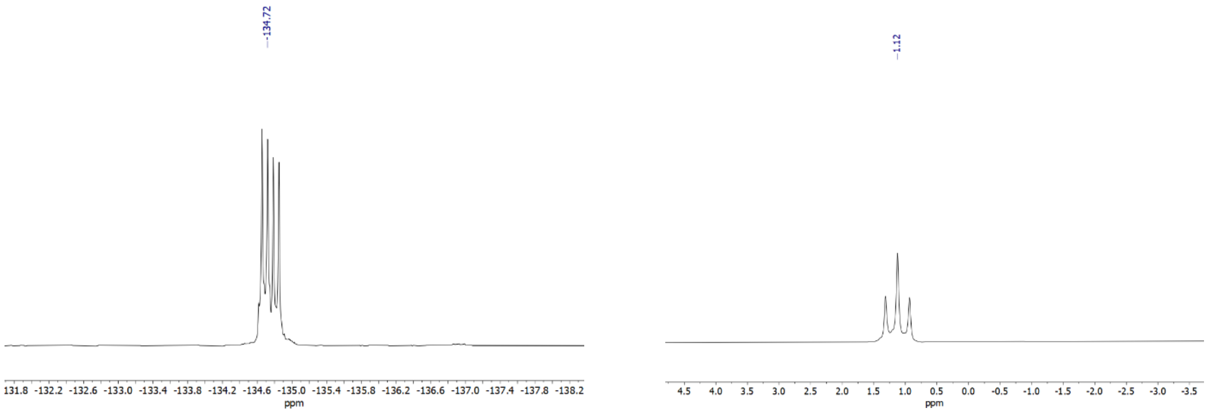

Figure S5.24. ^{19}F-NMR (left) and spectra ^{11}B-NMR (right) ($CDCl_3$) of **DIM-para**

BODIPY **DIM-meta**

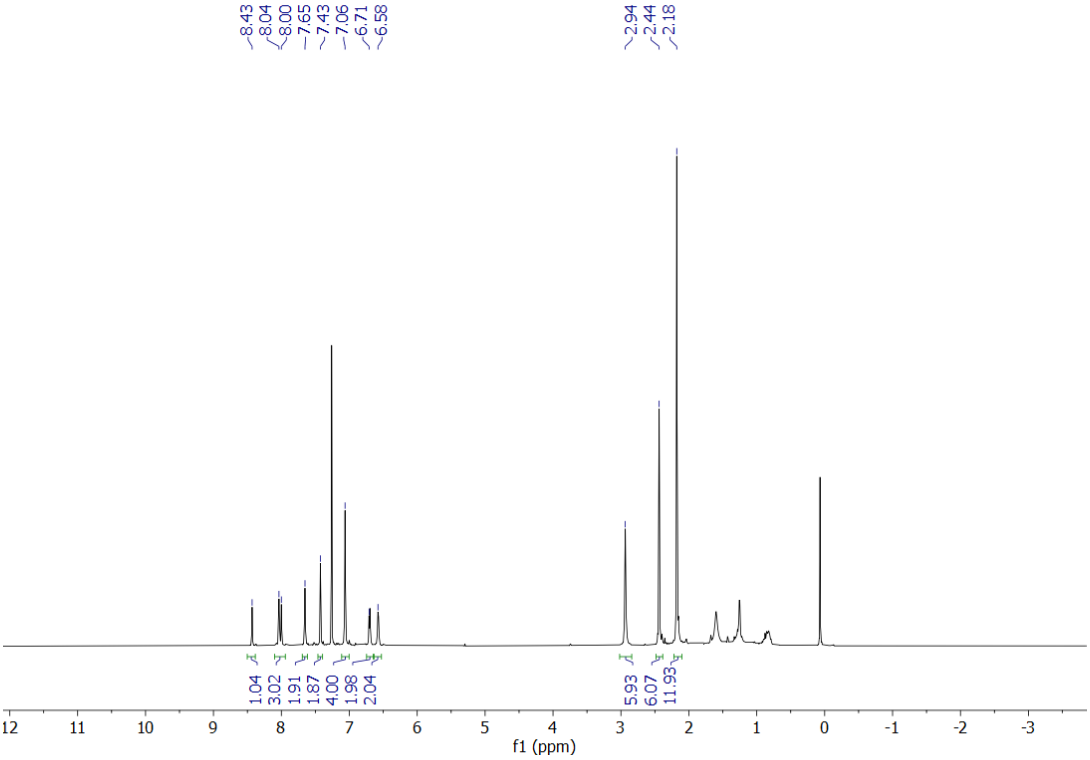

Figure S5.22. ^{1}H-NMR spectrum ($CDCl_3$) of **DIM-meta**

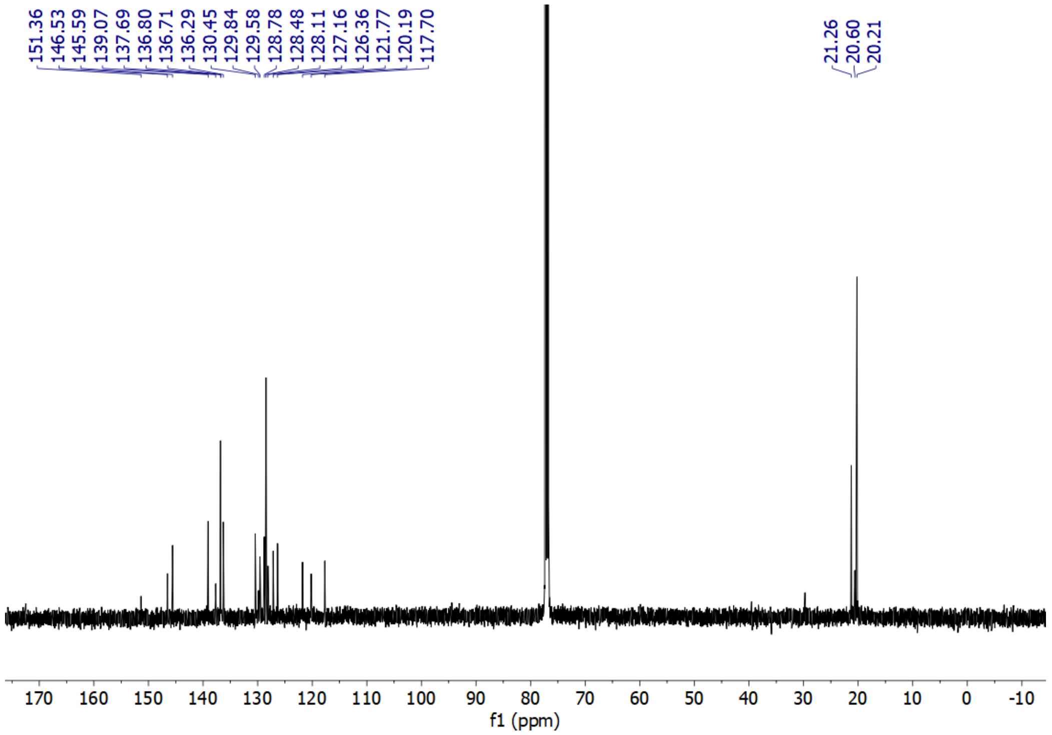

Figure S5.23. ^{13}C-NMR spectrum ($CDCl_3$) of **DIM-meta**

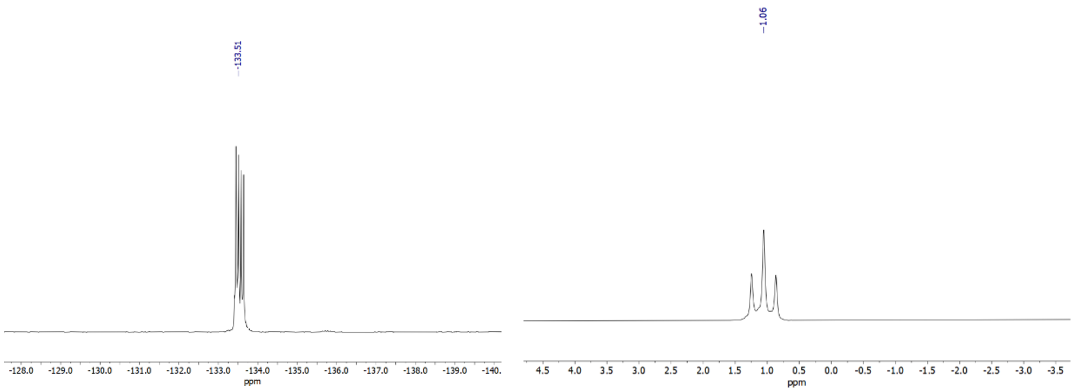

Figure S5.24. ^{19}F-NMR (left) and spectra ^{11}B-NMR (right) ($CDCl_3$) of **DIM-meta**

BODIPY **TRIM-para**

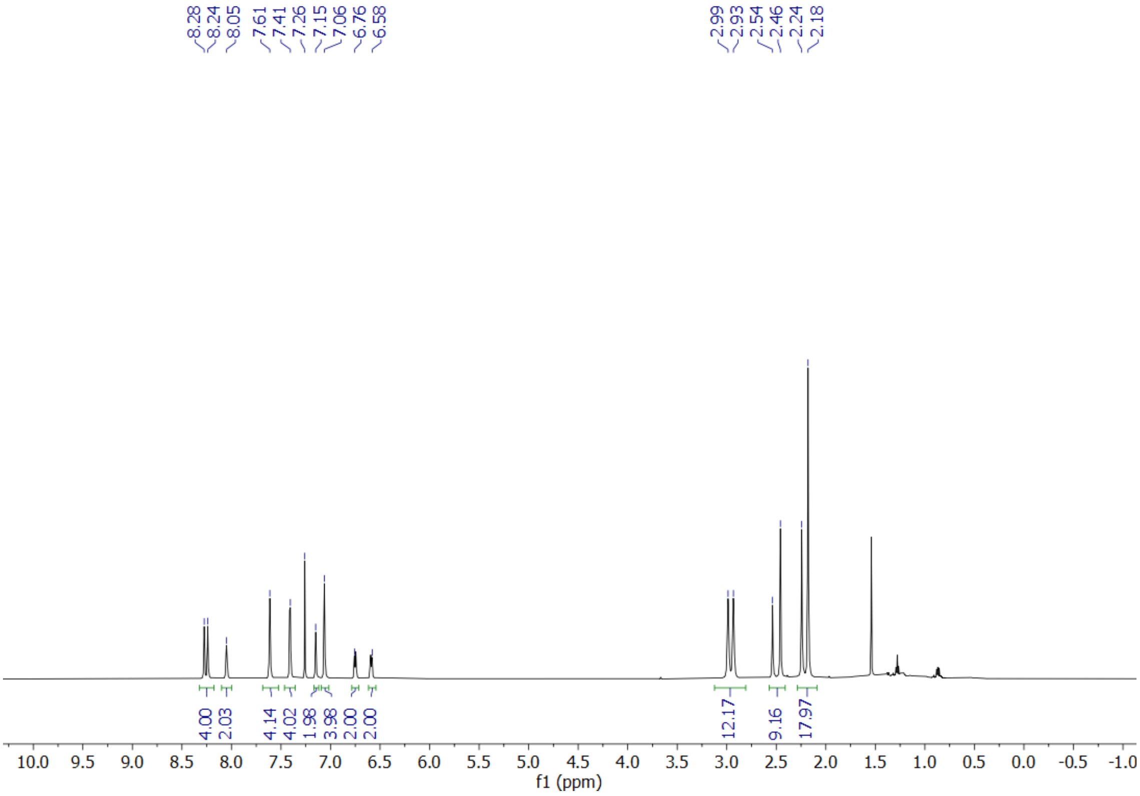

Figure S5.25. ^{1}H-NMR spectrum ($CDCl_3$) of **TRIM-para**

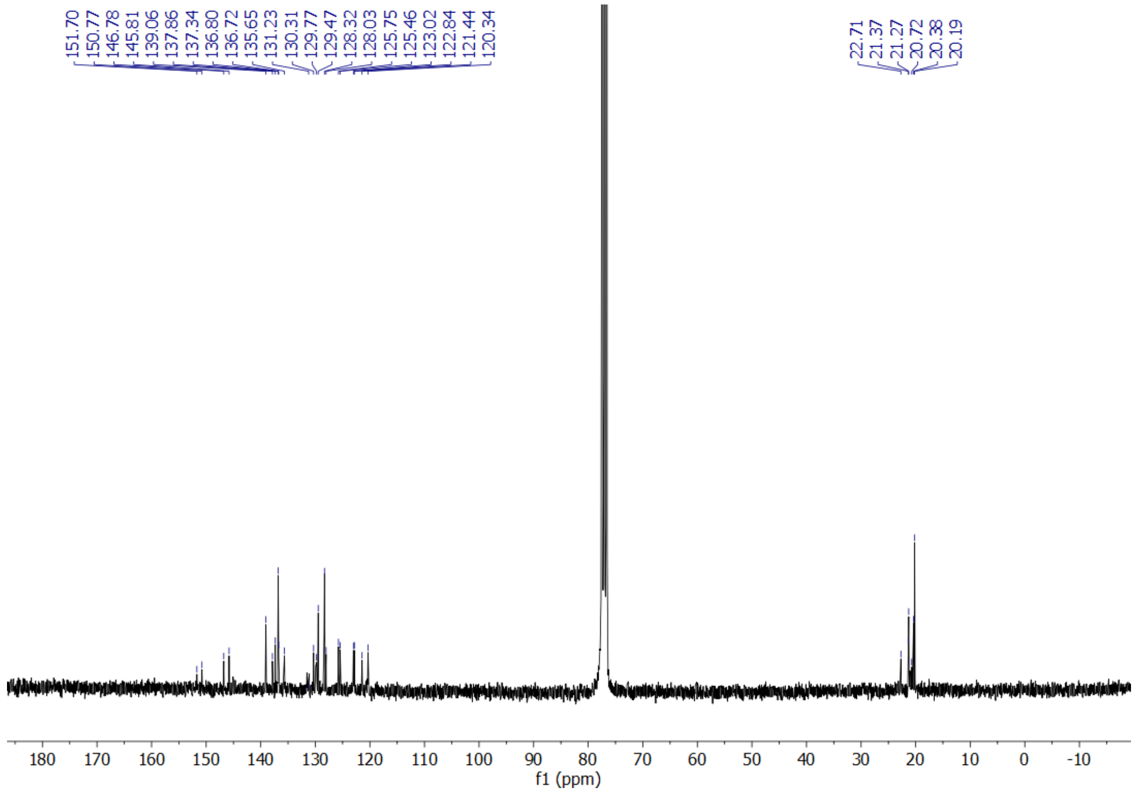

Figure S5.26. ^{13}C-NMR spectrum ($CDCl_3$) of **TRIM-para**

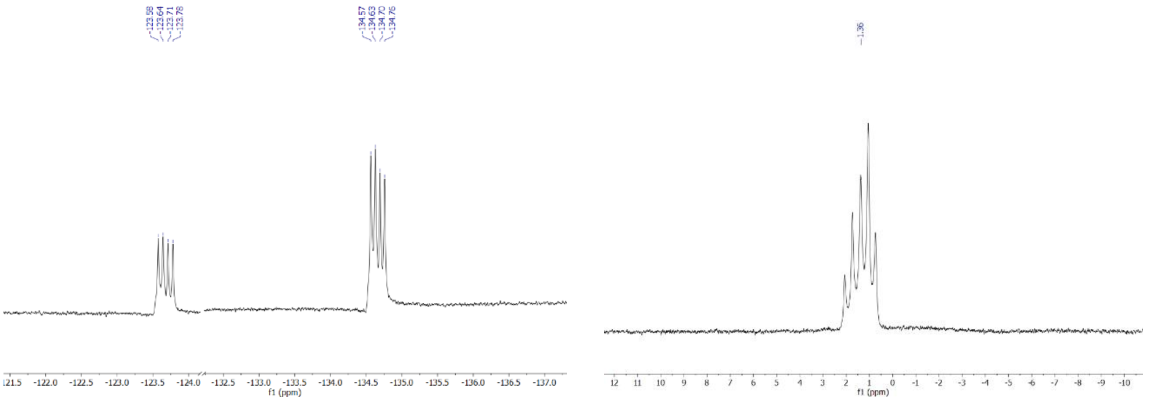

Figure S5.27. ^{19}F-NMR (left) [1]and spectra [1]B-NMR (right) ($CDCl_3$) of **TRIM-para**

BODIPY **<u>TRIM-meta</u>**

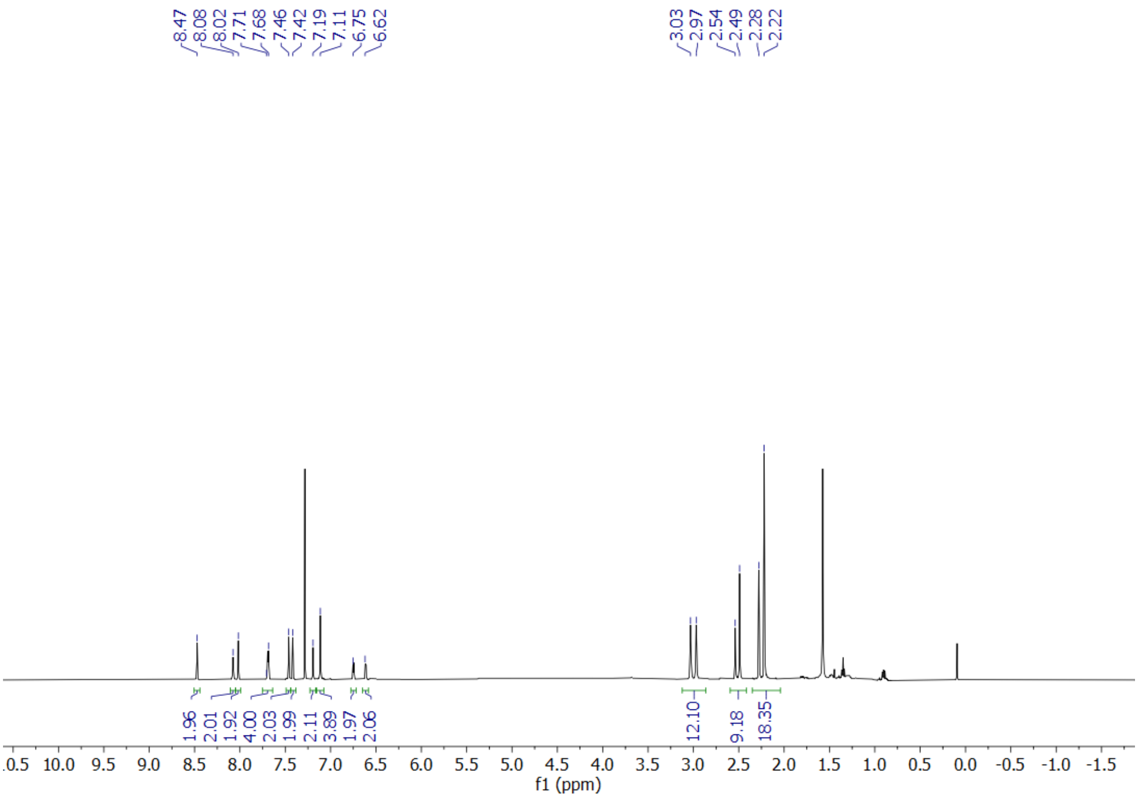

Figure S5.28. ^{1}H-NMR spectrum ($CDCl_3$) of **TRIM-meta.**

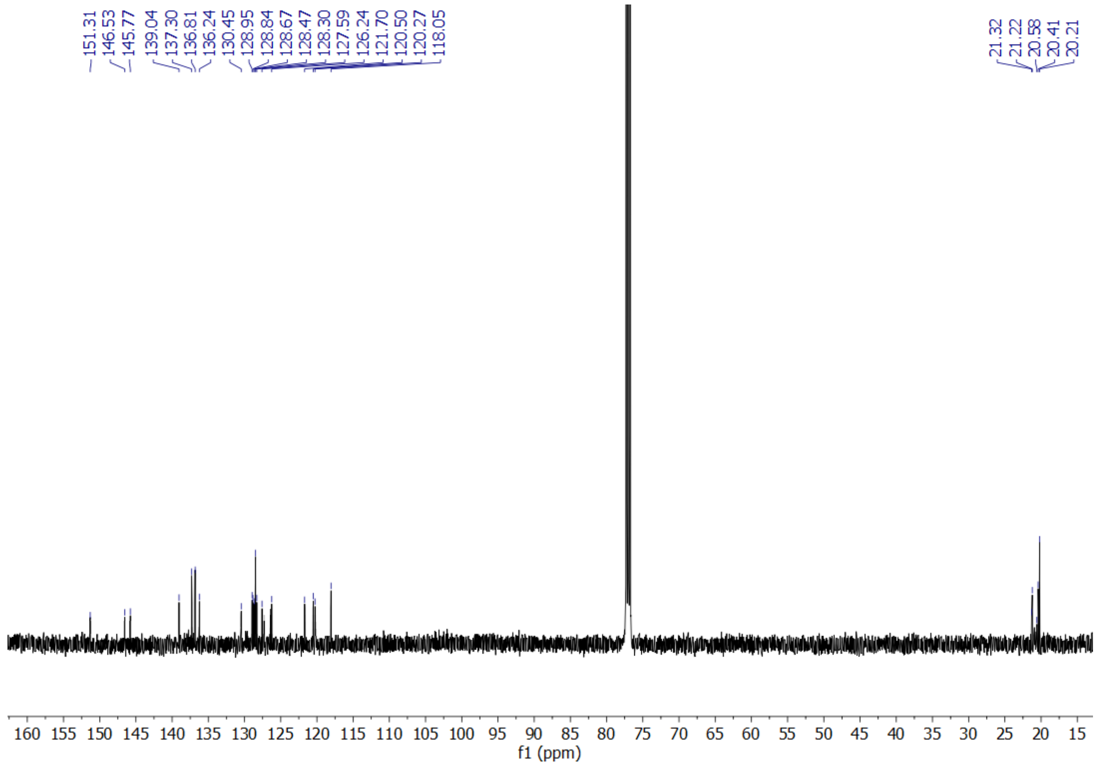

Figure S5.29. ^{13}C-NMR spectrum ($CDCl_3$) of **TRIM-meta**

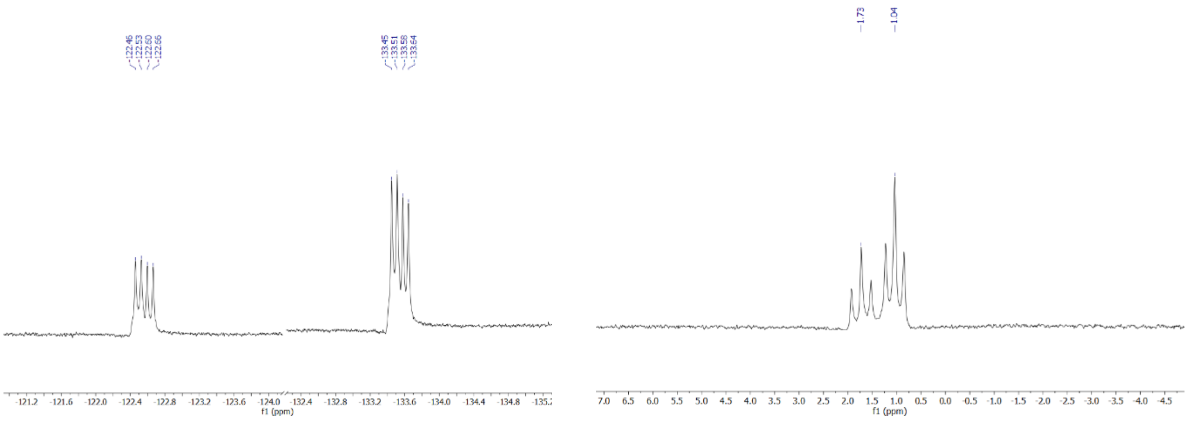

Figure S5.30. ^{19}F-NMR (left) ^{1}and spectra ^{1}B-NMR (right) ($CDCl_3$) of **TRIM-meta**

5. **Computational Studies**

All reported structures were optimized at DFT level using the B3LYP [7] functional and the standard 6-31G(d) basis set. Analytical harmonic frequencies were computed at the same level of theory to confirm the nature of the stationary points. NICS(0) values were calculated at the GIAO-B3lyp/6-31+G(d,p) level. TD-DFT calculations were carried out at the CAM-B3LYP/6-31+G(d,p) and considering the solvent (DCM) through the implicit Polarizable Continuum Model (PCM). All of the calculations were carried out by the methods implemented in Gaussian 16 package.[8]

Theorical structure and selected C-C bond lengths of **TRIM-para**

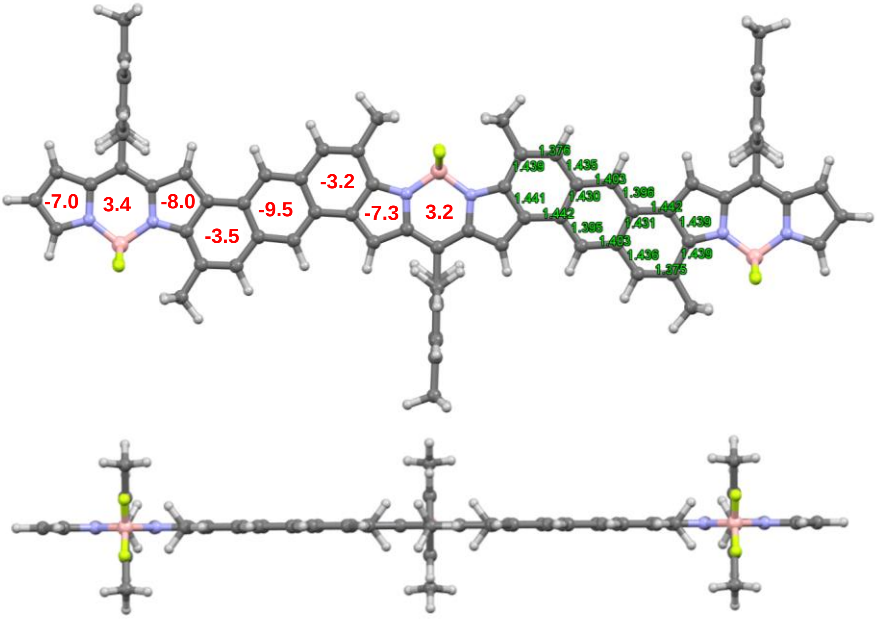

Figure S5.1. Front and side-view of **TRIM-para** theorical structure, calculated at B3LYP/6-31+G(d,p) level of theory.

Electrostatic potential surfaces of BODIPY-anthracene oligomers

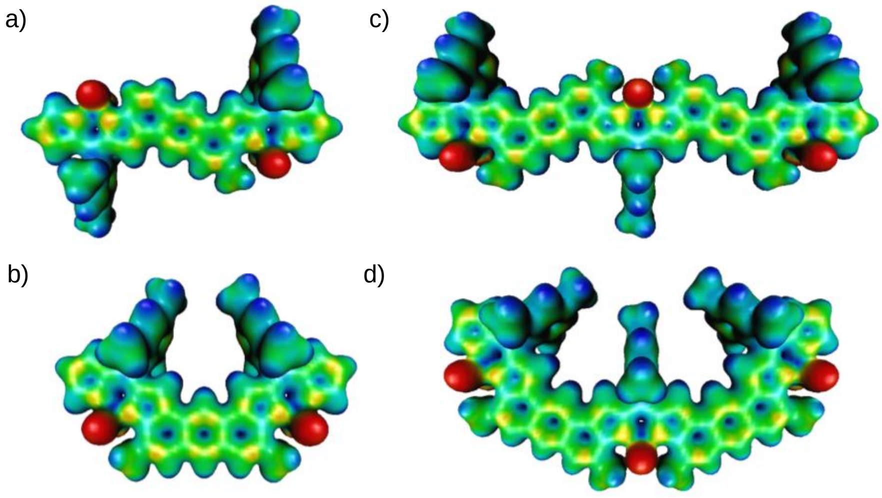

Figure S5.2. Electrostatic potential surfaces of (a) **DIM-para**, (b) **DIM-meta**, (c) **TRIM-para**. (d) TRIM-meta, calculated at B3LYP/6-31+G(d,p) level of theory.

Frontier molecular orbitals of BODIPY-anthracene oligomers

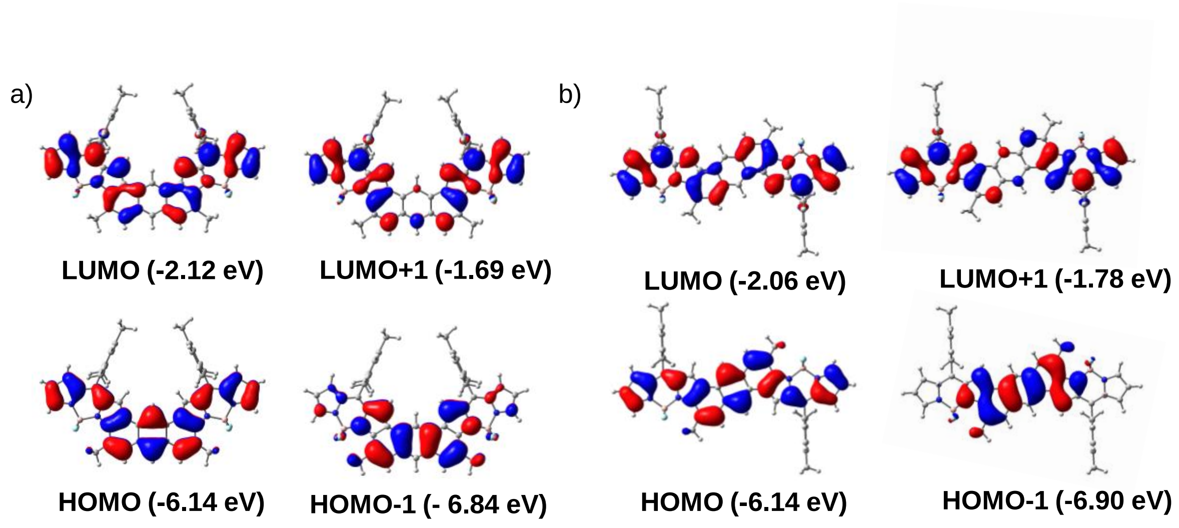

Figure S5.3. Calculated frontier molecular orbital profiles of a) **DIM-meta** and b) **DIM-para**.

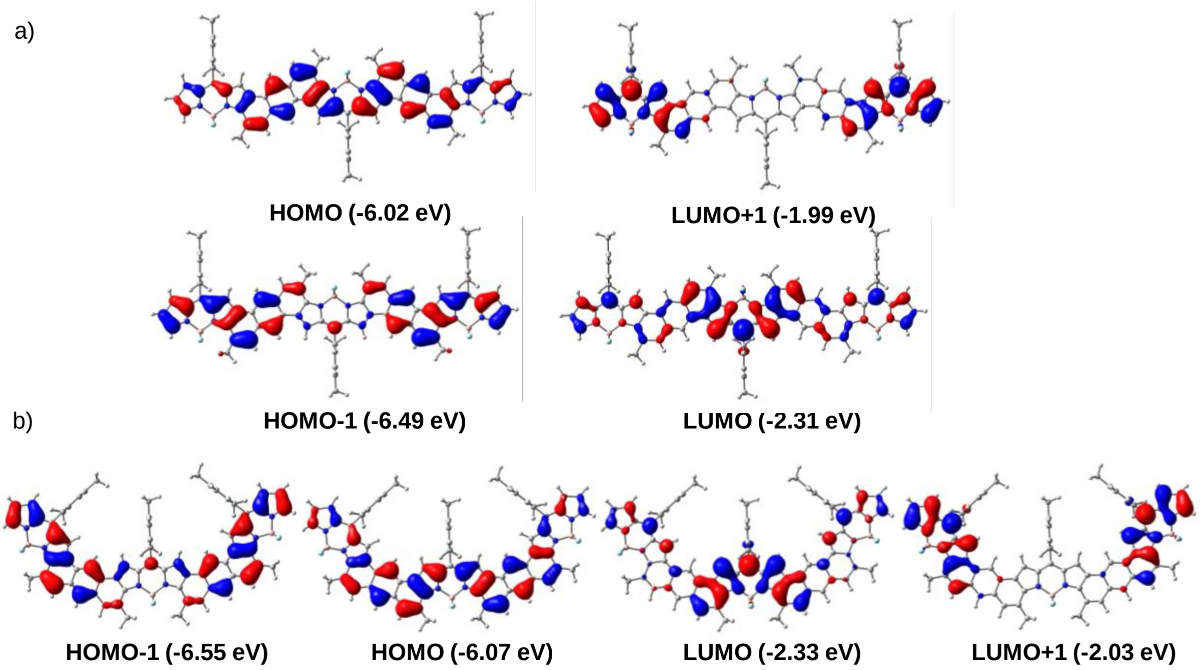

Figure S5.4. Calculated frontier molecular orbital profiles of a) **TRIM-para** and b) **TRIM-meta**.

TD-DFT calculations

Compd.	Energy (nm)	f[a]	Orbitals[b] (coefficient)
**DIM-para**	521	1.92	H→L (70 %)
**DIM-meta**	522	1.32	H→L (70%)
	445	0.37	H→L+1 (60 %)
**TRIM-para**	607	3.41	H→L (65 %), H-1→L+1 (20%)
**TRIM-meta**	606	2.21	H→L (65 %), H-1→L+1 (20%)
	485	0.90	H→L+1 (47 %),H-1→L (38%)

Table S5.1. Selected transition properties of BODIPY-anthracene oligomers calculated at CAM-B3LYP/6-31+G(d,p) level of theory. [a]Oscillator strength. [b]MOs involved in the transitions (H and L denoting HOMO and LUMO)

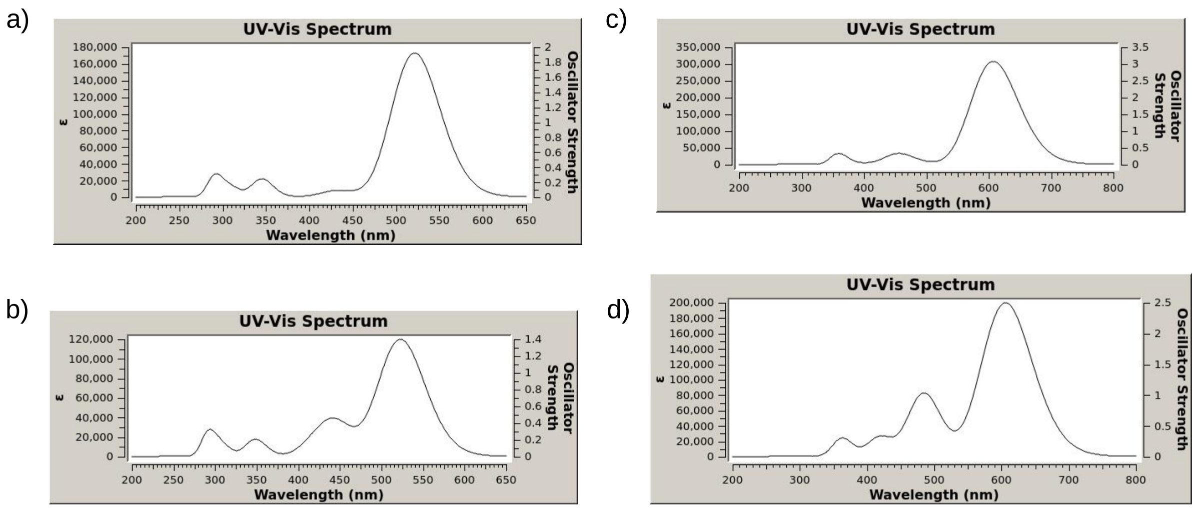

Figure S5.5. Calculated spectra of a) **DIM-para**, b) **DIM-meta**, c) **TRIM-para**, d) **TRIM-meta** oligomers calculated at CAM-B3LYP/6-31+G(d,p) level of theory.

6. Photophysical characterization

	Solvent	λ_{max} [nm]	ε / 10^5 [$M^{-1}cm^{-1}$]	Φ_f	τ [ns]
**DIM-para**	Toluene	694	2.77	0.56	2.90
	THF	685	2.26	0.35	2.27
	DCM	685	1.77	0.36	2.35
**DIM-meta**	Toluene	691	2.10	0.59	3.31
	THF	686	1.33	0.17	1.91
	DCM	686	2.39	0.18	1.84
**TRIM-para**	Toluene	807	4.97	0.26	2.22
	THF	796	3.77	0.20	1.66
	DCM	794	4.62	0.22	1.97
**TRIM-meta**	Toluene	802	5.08	0.20	2.59
	THF	796	3.67	0.13	1.79
	DCM	794	3.47	0.14	2.05

Table S6.1. Absorption maxima (λ_{max}), molar extinction coefficients (at λ_{max}), fluorescence quantum yield (Φ_f) [a] and lifetime (τ) of **DIM-para**, **DIM-meta**, **TRIM-para** and **TRIM-meta** in different solvents. [a] Φ_f of **DIM-para**, **DIM-meta** calculated *vs*. ZnPc in toluene (0.34) and of **TRIM-para**, **TRIM-meta** calculated *vs*. TATBP derivative in toluene (0.10).

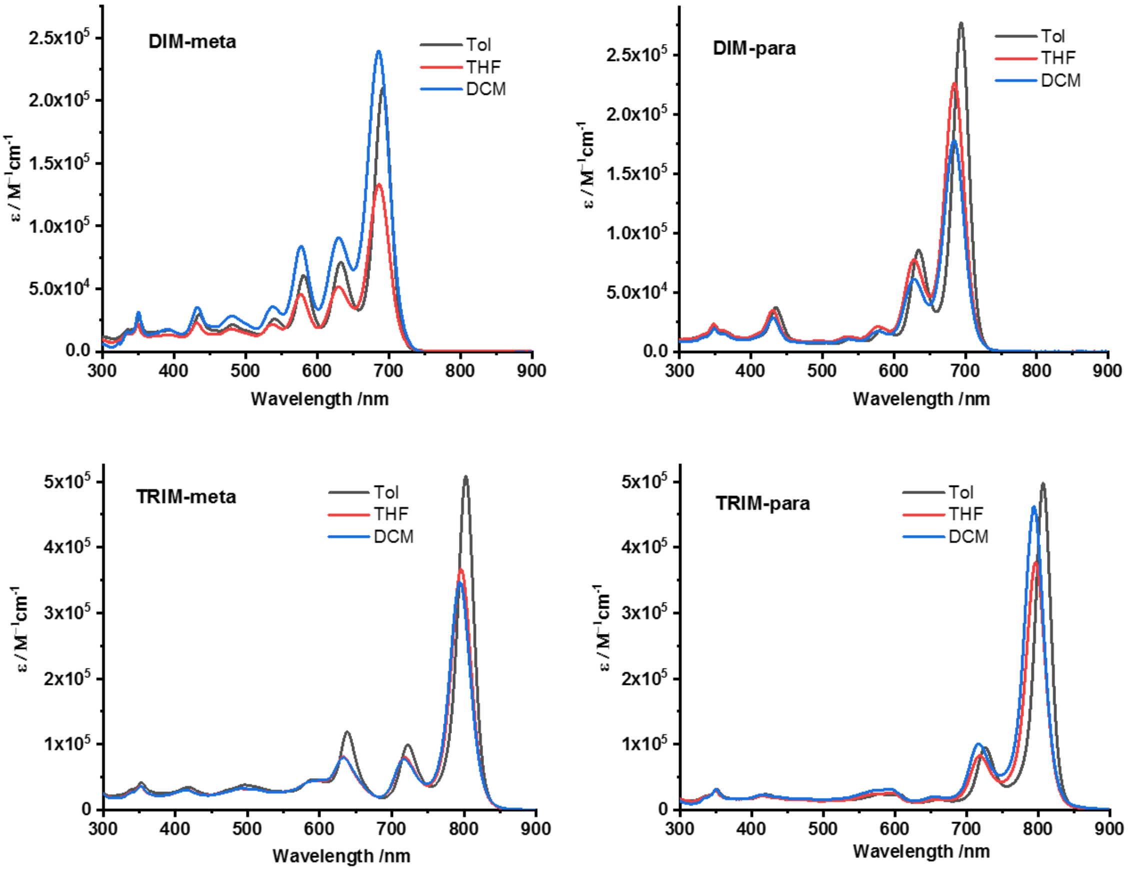

Figure S6.1. Absorption spectra of **DIM-meta, DIM-para, TRIM-meta** and **TRIM-para** recorded in toluene, THF and DCM at room temperature.

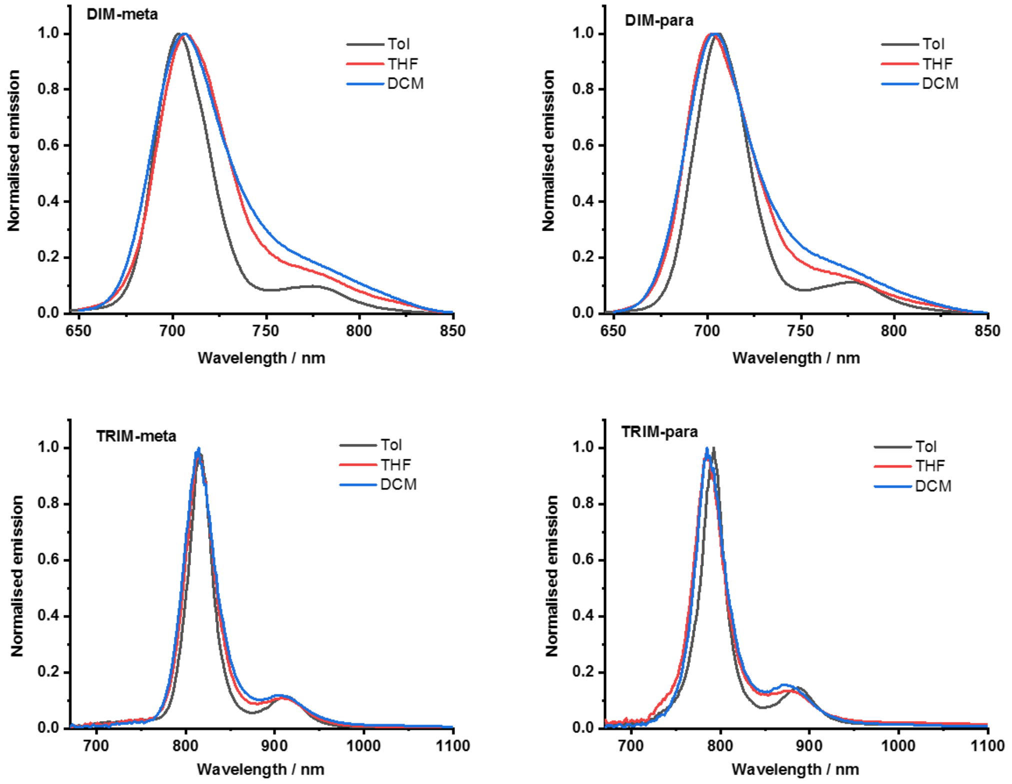

Figure S6.2. Normalised fluorescence spectra of **DIM-meta, DIM-para** (λ_{ex} – 630 nm) **TRIM-meta** and **TRIM-para** (λ_{ex} – 650 nm) recorded in toluene, THF and DCM at room temperature.

7. Transient absorption studies

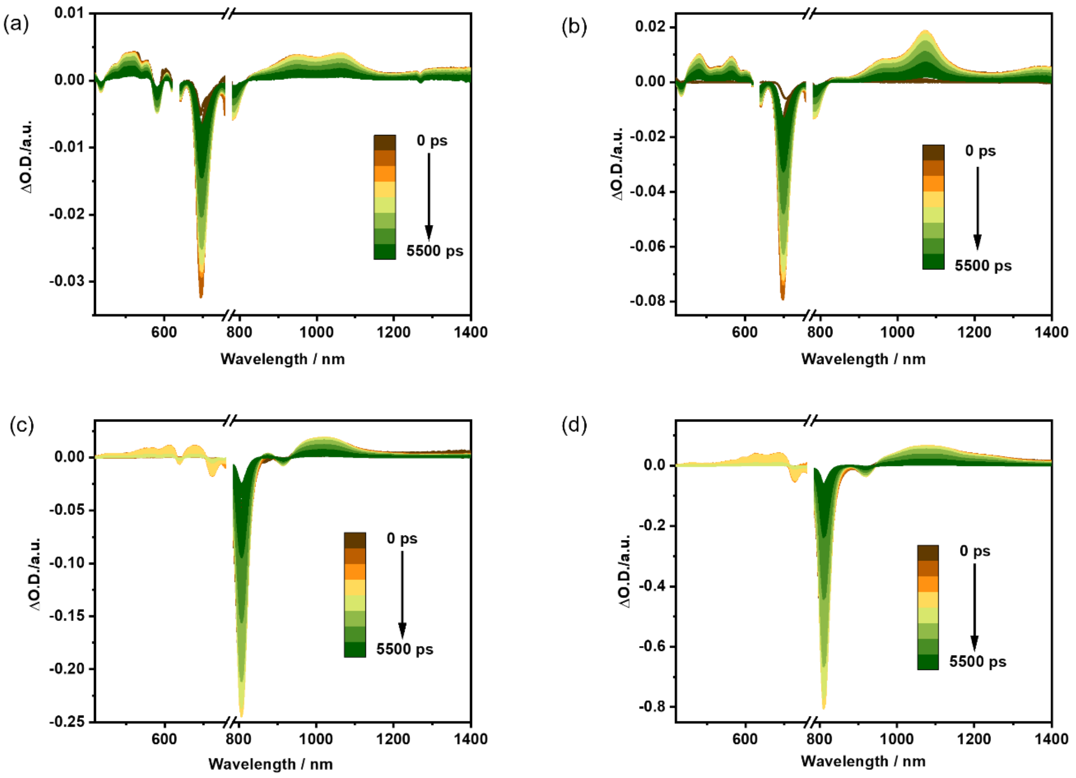

Figure S8.1. Femtosecond differential absorption spectra of a) **DIM-meta**, b) **DIM-para**, c) **TRIM-meta** and d) **TRIM-para** measured in argon-purged toluene at room temperature, at time delays between 0 – 5500 ps.

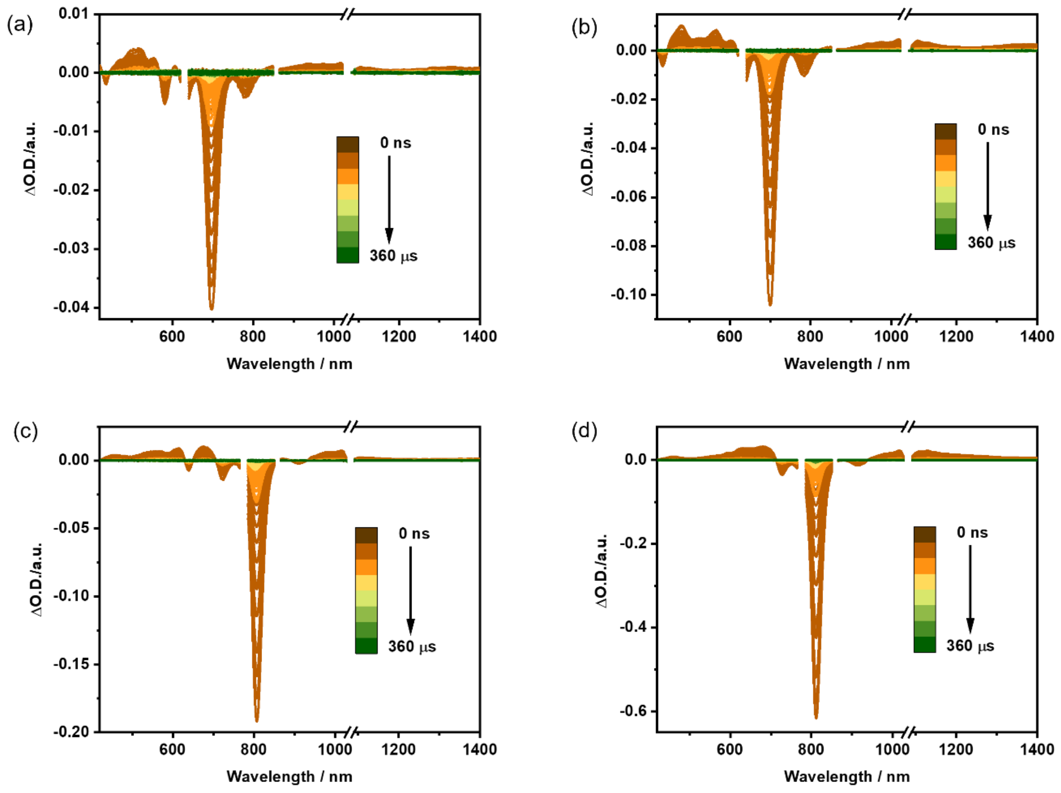

Figure S8.2. Nanosecond differential absorption spectra of a) **DIM-meta**, b) **DIM-para**, c) **TRIM-meta** and d) **TRIM-para** measured in argon-purged toluene at room temperature, at time delays between 1 ns and 360 μs.

8. **Electrochemistry**

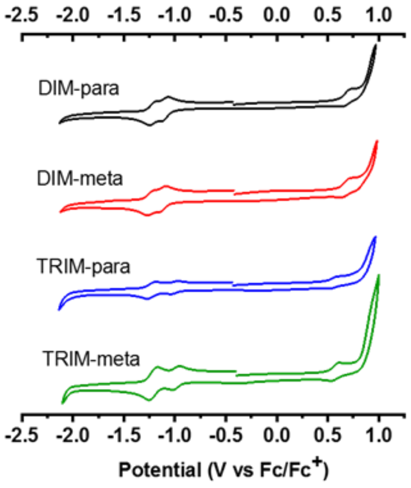

Figure S8.1. Cyclic voltammograms of **DIM-meta**, **DIM-para**, **TRIM-meta** and **TRIM-para** in 0.1 M TBAPF$_6$ in DCM versus Fc/Fc$^+$ at room temperature.

9. **References**

[1] N. R. Babij, E. O. McCusker, G. T. Whiteker, B. Canturk, N. Choy, L. C. Creemer, C. V. D. Amicis, N. M. Hewlett, P. L. Johnson, J. A. Knobelsdorf, F. Li, B. A. Lorsbach, B. M. Nugent, S. J. Ryan, M. R. Smith and Q. Yang, *Org. Process Res. Dev*., 2016, **20**, 661.

[2] J. R. Lakowicz, *Principles of fluorescence spectroscopy*, 3rd ed. Springer, New York, 2006.

[3] A. M. Brouwer, *Pure Appl. Chem.*, 2011, **83**, 2213.

[4] The authors thank X-Ray laboratory for the crystallographic support.

[5] J. J. Snellenburg, S. P. Laptenok, R. Seger, K. M. Mullen and I. H. van Stokkum, J. Stat. Softw., 2012, 49, 1-22.

[6] (a) Y. Hayashi, S. Yamaguchi, W. Y. Cha, D. Kim, and H. Shinokubo, *Org. Lett.* 2011, **13**, 2992; (b) G. R. Kiel, M. S. Ziegler and T. D. Tilley, *Angew.Chem. Int.Ed.* 2017, **56**,4839

[7] (a) C. Lee, W. Yang, R. G. Parr, *Phys. Rev. B* 1988, **37**, 785–789. (b) A. D. Becke, *J. Chem. Phys.* 1993, **98**, 5648. (c) W. Kohn, A. D. Becke, R. G. Parr, *J. Phys. Chem.* 1996, **100**, 12974–12980.

[8] Gaussian 16, Revision C.01; Frisch, M. J.; Trucks, G. W.; Schlegel, H. B.; Scuseria, G. E.; Robb, M. A.; Cheeseman, J. R.; Scalmani, G.; Barone, V.; Petersson, G. A.; Nakatsuji, H.; Li, X.; Caricato, M.; Marenich, A. V.; Bloino, J.; Janesko, B. G.; Gomperts, R.; Mennucci, B.; Hratchian, H. P.; Ortiz, J. V.; Izmaylov, A. F.; Sonnenberg, J. L.; Williams-Young, D.; Ding, F.; Lipparini, F.; Egidi, F.; Goings, J.; Peng, B.; Petrone, A.; Henderson, T.; Ranasinghe, D.; Zakrzewski, V. G.; Gao, J.; Rega, N.; Zheng, G.; Liang, W.; Hada, M.; Ehara, M.; Toyota, K.; Fukuda, R.; Hasegawa, J.; Ishida, M.; Nakajima, T.; Honda, Y.; Kitao, O.; Nakai, H.; Vreven, T.; Throssell, K.; Montgomery, J. A., Jr.; Peralta, J. E.; Ogliaro, F.; Bearpark, M. J.; Heyd, J. J.; Brothers, E. N.; Kudin, K. N.; Staroverov, V. N.; Keith, T. A.; Kobayashi, R.; Normand, J.; Raghavachari, K.; Rendell, A. P.; Burant, J. C.; Iyengar, S. S.; Tomasi, J.; Cossi, M.; Millam, J. M.; Klene, M.; Adamo, C.; Cammi, R.; Ochterski, J. W.; Martin, R. L.; Morokuma, K.; Farkas, O.; Foresman, J. B.; Fox, D. J. Gaussian, Inc., Wallingford CT, 2016.
